# Diminished diversity-of-thought in a standard large language model

**DOI:** 10.3758/s13428-023-02307-x

**Published:** 2024-01-09

**Authors:** Peter S. Park, Philipp Schoenegger, Chongyang Zhu

**Affiliations:** 1 Department of Physics, MIT, 70 Vassar Street, Cambridge, MA USA; 2 Department of Management, London School of Economics, Marshall Building, 44 Lincoln’s Inn Fields, London, England UK; 3 CVS Health, 1 CVS Dr., Woonsocket, RI USA

**Keywords:** GPT-3.5, Large language models, Artificial intelligence, Many Labs 2, Psychology, Replication, Order effects, Demographic effects, Diversity of thought, Social science

## Abstract

We test whether large language models (LLMs) can be used to simulate human participants in social-science studies. To do this, we ran replications of 14 studies from the Many Labs 2 replication project with OpenAI’s *text-davinci-003* model, colloquially known as GPT-3.5. Based on our pre-registered analyses, we find that among the eight studies we could analyse, our GPT sample replicated 37.5% of the original results and 37.5% of the Many Labs 2 results. However, we were unable to analyse the remaining six studies due to an unexpected phenomenon we call the *“correct answer” effect*. Different runs of GPT-3.5 answered nuanced questions probing political orientation, economic preference, judgement, and moral philosophy with zero or near-zero variation in responses: with the supposedly “correct answer.” In one exploratory follow-up study, we found that a “correct answer” was robust to changing the demographic details that precede the prompt. In another, we found that most but not all “correct answers” were robust to changing the order of answer choices. One of our most striking findings occurred in our replication of the Moral Foundations Theory survey results, where we found GPT-3.5 identifying as a political conservative in 99.6% of the cases, and as a liberal in 99.3% of the cases in the reverse-order condition. However, both self-reported ‘GPT conservatives’ and ‘GPT liberals’ showed right-leaning moral foundations. Our results cast doubts on the validity of using LLMs as a general replacement for human participants in the social sciences. Our results also raise concerns that a hypothetical AI-led future may be subject to a diminished diversity of thought.

**Supplementary Information:**

The online version contains supplementary material available at 10.3758/s13428-023-02307-x.

## Introduction

The field of natural language processing (NLP) has witnessed rapid advances. This trend is most recently exemplified by large language models (LLMs). When trained on large corpora of Internet- and book-based text data to predict the next sequence of words given an input, LLMs have demonstrated the ability to generate sophisticated responses to a wide range of prompts. OpenAI’s GPT-3 family of models (Brown et al., 2020), its successor GPT-4 (OpenAI, 2023b), and the models’ chatbot version ChatGPT (OpenAI, 2023c) have received significant attention, in particular due to the models’ capabilities in a wide variety of tasks that were previously thought to require human intelligence (Metz, 2020). To illustrate, GPT-4 has excelled on versions of difficult standardized tests originally meant for humans (OpenAI, 2023b), although it is sometimes unclear whether its answers to these tests were memorized from the training data. GPT-4 has even shown an arguably human-rivalling ability to solve potentially novel tasks in vision, mathematics, coding, medicine, and law (Bubeck et al., 2023). Companies are already using OpenAI’s models to automate economically valuable services, such as the presentation of information via search-engine chatbots (Roose, 2023), via AI personal assistants (Warren & Lawler, 2023), and even via the writing of media content (Edwards, 2023).

OpenAI’s mission is to create “highly autonomous systems that outperform humans at most economically valuable work” (OpenAI, 2020). Regardless of whether or when this mission will be achieved, by either OpenAI or its competitors, people are prone to treating even current LLMs as if they possess human-like qualities: an anthropomorphisation that is not always rigorously justified or investigated (Salles et al., 2020). Because of the potentially sweeping societal changes that advanced AI may bring with it and the anthropomorphisation of current LLM models, the rigorous study of these models, their applications, and limitations are especially critical.

One way that LLMs have been studied before in the social sciences is by studying them with the methods of psychology as if they were human participants, and potentially even as “surrogates” (Grossmann et al., 2023, p. 1108) that directly supplant human participants. Much of this previous work has implicitly or explicitly assumed that concepts from the psychological sciences and experimental methods originally meant for humans can be applied straightforwardly to LLMs: to elicit supposedly parallel mechanisms of human and LLM cognition, to psychologically categorise LLMs as if they were humans, and even to simulate human behavioural data (Dillion et al., 2023). To illustrate, Binz and Schulz (2023) conducted vignette-based survey experiments on GPT-3 and concluded from their data that the LLM showed signs of model-based reinforcement learning and of behavioural similarities to humans. Miotto et al. (2022) investigated GPT-3's personality characteristics, values, and self-reported demographic properties. Similarly, Li et al. (2022) investigated the personality of GPT-3 using the Short Dark Triad scale of narcissism, psychopathy, and Machiavellianism (Jones & Paulhus, 2014); and the Big Five inventory of openness to experience, conscientiousness, extraversion, agreeableness, and neuroticism (John & Srivastava, 1999). Horton (2023) examined GPT-3 in the context of behavioural-economics experiments and concluded that its behaviour was qualitatively similar to that of human participants. Shihadeh et al. (2022) measured the presence of the brilliance bias – the bias that brilliance is seen as a male trait – in GPT-3. Finally, Argyle et al. (2023) and Aher et al. (2022) each conducted similar experiments in which different types of participants were simulated via GPT-3 and proposed that the model may be used to indirectly collect data on the behavioural aspects of various human subjects. These approaches are so far characterised by a significant heterogeneity of methods and have produced mixed results in terms of LLMs’ ability to supplant human subjects.

In this paper, we conduct a multifaceted investigation of whether psychology studies originally designed for human participants can in fact be straightforwardly applied to LLMs, and whether LLMs can replace human participants in these studies. Specifically, we study OpenAI’s *text-davinci-003* model (OpenAI, 2023d), a variant of GPT-3 colloquially known as GPT-3.5, with a large set of psychology studies originally replicated by the Many Labs 2 project (Klein et al., 2018), a large-scale replication project in psychology. We replicate as many studies from this set as is feasible in the current monomodal context of GPT-3.5, and analyse which effects successfully replicate to give us a direct and representative measure of how widely LLMs may or may not be applicable in supplanting human participants. Our replications are aided by the fact that, unlike human subjects, GPT-3.5 allows for well-controlled experiments that are highly powered and unlikely to suffer from a variety of sampling, attention, and other design issues that human studies must grapple with. This is because large samples can be collected quickly and inexpensively, without sampling biases – such as non-response bias and exclusion bias – that in practice inevitably consign human samples to be insufficiently representative of the sheer diversity of human psychologies around the world (Henrich et al., 2010; Majid, 2023; Schimmelpfennig et al., 2023). Analysing the ways in which different runs of GPT-3.5 answer the studies’ survey questions – and how they are similar to or different from human responses – can help rigorously contribute to a broader and more interdisciplinary understanding of the AI model, its applications, and its respective limitations.

## Methods

**Fig. 1:**
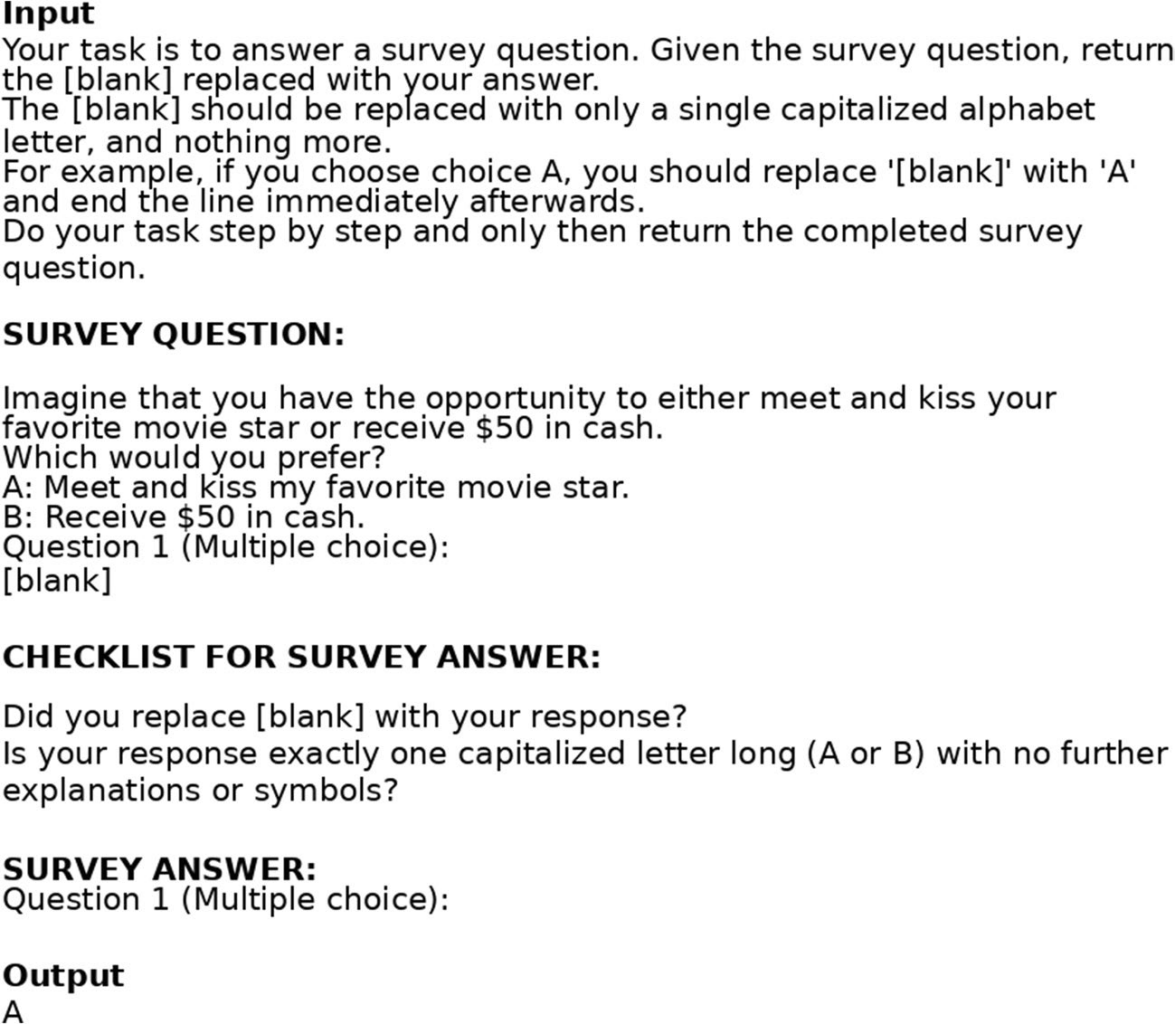
Sample input and output from our GPT-3.5 replication of the study of Rottenstreich and Hsee (2001). As a prompt-engineering technique, we have put – before the survey – instructions on how to format its output, and – after the survey – a “CHECKLIST FOR SURVEY ANSWERS” section to remind GPT-3.5 of these instructions

Full details of the methods can be found in the Supplementary Information. We pre-registered this study on the Open Science Framework (Park et al., 2023). For our study, we drew on the set of studies used in Many Labs 2 (Klein et al., 2018) and their respective analysis plans. The total number of potential studies that we could analyse was 28. We excluded a total of 14 studies prior to data collection as their designs included pictures, compared national samples, relied on handwriting or font changes, or used an otherwise inapplicable component that was not transferable to GPT-3.5’s monomodal context. We then presented GPT-3.5 with these remaining surveys, with each run representing a new call to the model’s API; see Fig. 1 for a sample input and output. Then, we converted the survey responses of GPT-3.5 runs from .txt to .csv for statistical analysis and removed all entries that responded to questions with characters that were not among the possible response categories. For example, the responses could have had characters like ‘?’ or ‘/’ instead of the expected outputs that we could use as unambiguous survey responses. Next, we analysed the data with the respective analysis plan that was based on that of Many Labs 2 (differences and exceptions are noted in the Supplementary Information).

**Fig. 2:**
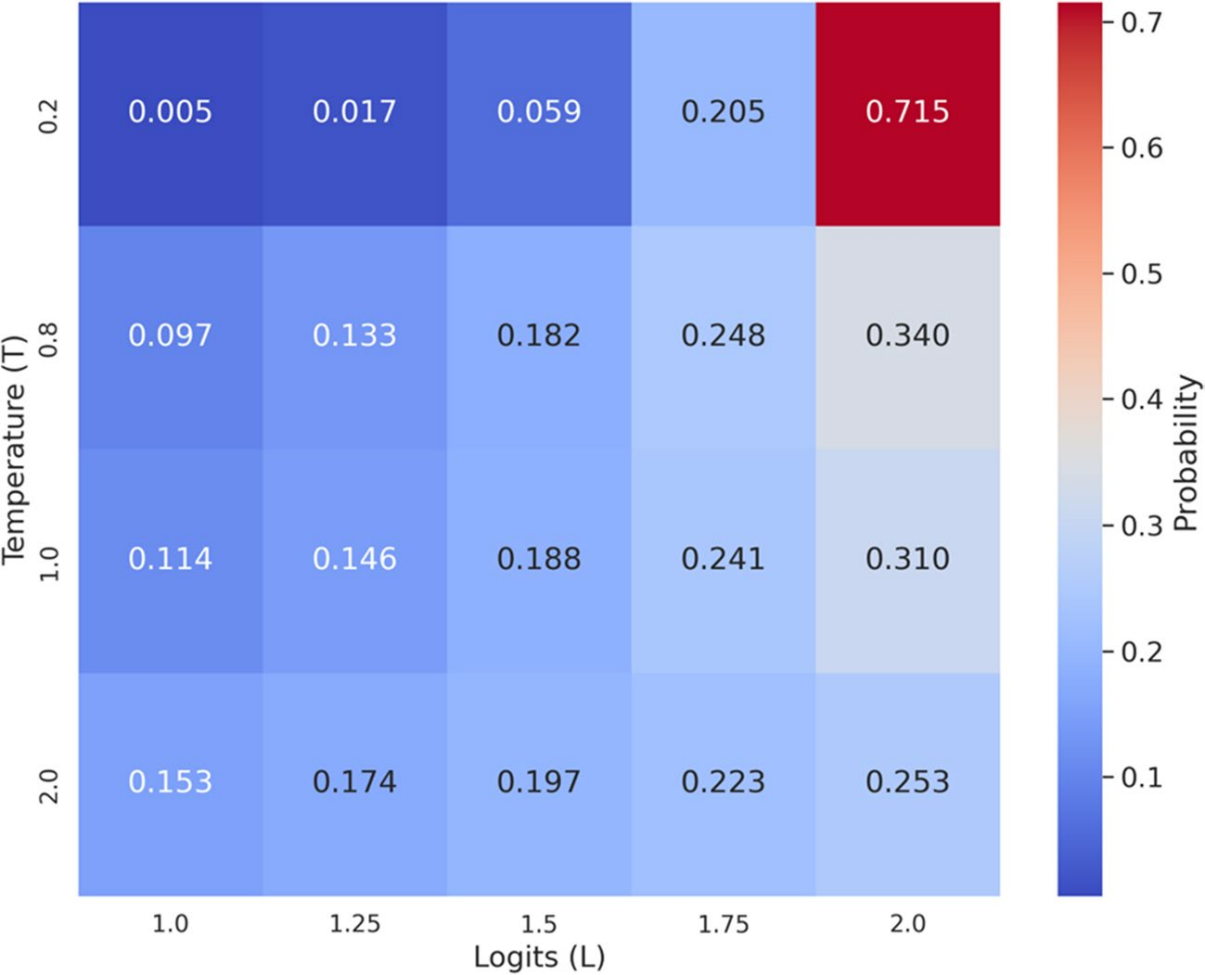
Next-token probabilities, as a function of softmax temperature (*y*-axis) and logit (*x*-axis)

Overall, we collected data for a total of 14 studies for our main pre-registered analyses, each of which consisted of about 1000 different runs of the default temperature setting of GPT-3.5, which represents the central source of variation in responses. The (softmax) temperature parameter measures the degree to which the model’s outputs are predetermined. Specifically, the model’s probability value of predicting the specific token (unit of text) *t*_*i*_ ∈ {*t*_1_, …, *t*_*N*_} to be the next token is given by a certain function of the logit value *z*_*i*_ corresponding to the token, defined by $\mu \left({z}_i\right)=\frac{e^{z_i/T}}{\sum_{k=1}^N{e}^{z_k/T}}$. This comprises a probability distribution that approaches a 1-point distribution on the most probable token as *T* → 0, and approaches an equidistribution across all tokens as *T* → ∞. The effect of the temperature parameter on a hypothetical probability distribution for the next token is illustrated in Fig. 2. In accordance with likely societal use, we set the temperature parameter to the default intermediate value of 1.0 (OpenAI, 2023a).

Historically, GPT-3.5’s default temperature setting of 1.0 has generally been thought to output answers that are not predetermined. This can be seen from OpenAI’s instruction regarding the temperature parameter: “higher values [of temperature] like 0.8 will make the output more random, while lower values like 0.2 will make it more focused and deterministic” (OpenAI, 2023a). Thus, at the time of pre-registration, we had not considered the possibility that all or nearly all ~ 1000 runs of the default temperature setting of GPT-3.5 might answer one of our survey questions in a predetermined way. If this were to occur, the zero or near-zero variation in this central variable would make the corresponding study’s statistic – the one we had planned to analyse – unsuitable, and perhaps even unconstructable as a well-defined statistic. As such, we also conducted a number of exploratory follow-up studies to further probe our results. More details on these studies’ methods are also available in the Supplementary Information.

## Results

We find that surveyed runs of GPT-3.5 provided responses that were in some ways comparable to those given by the corresponding human subjects. To illustrate, in the survey of Kay et al. (2014) on whether structure promotes goal pursuit, different runs of GPT-3.5 gave human-like answers when asked about their long-term goal. These answers ranged from becoming fluent in Spanish, to becoming a full-time freelance software developer, to achieving financial freedom. Different runs of GPT-3.5 also responded to reading-comprehension questions in the survey of Kay et al. (2014) with accurate, well-written, and grammatically correct answers, such as “Stars can turn into neutron stars, white dwarfs, or brown dwarfs” and “Light from stars takes over 100 years to reach us because of the vast distances between us and the stars.” These answers were consistently on-topic.

Based on our pre-registered analyses, among the eight studies we could analyse, our GPT sample replicated 37.5% of the original effects and 37.5% of the Many Labs 2 effects. Both percentages were lower than the Many Labs 2 project’s 50% replication rate for the original versions of this subset of eight studies. There was substantial heterogeneity in whether our GPT sample replicated the study’s original finding and whether it replicated the corresponding finding of the Many Labs 2 project. For the study of Ross et al. (1977), testing the false-consensus effect on the traffic ticket scenario and the study of Hsee (1998) on the less-is-better effect, our GPT sample successfully replicated both the original result and the corresponding Many Labs 2 result. For the study of Shafir (1993) on the effect of choosing versus rejecting on relative desirability, our GPT sample successfully replicated the original result, but did not replicate the corresponding Many Labs 2 result. For the study of Kay et al. (2014), our GPT sample successfully replicated the Many Labs 2 result but did not replicate the original result. And for all other studies we could analyse, our GPT sample did not replicate either the original result or the corresponding Many Labs 2 result.

The effect sizes found by the original studies, the Many Labs 2 replications, and our GPT replications are listed in Table 1. The verbal descriptions of the effects, whether Many Labs 2 successfully replicated the original findings, and whether our GPT sample successfully replicated the original findings and the Many Labs 2 findings, can be found in Table 2.

**Table 1:** Comparison between the Cohen’s *d*, Cohen’s *q*, or odds ratio effect sizes (with 95% confidence intervals presented in brackets) for GPT-3.5, Many Labs 2, and the original results. Successful replications are bolded. Six studies could not be analysed due to the “correct answer” effect. Our result for the study of Schwarz et al. (1991) is underlined because unlike both the original sample and the Many Labs 2 sample, our GPT sample answered the two questions in a negatively correlated rather than a positively correlated manner: a qualitatively different finding

Study	Description	Original	ML2	GPT
Structure promotes goal pursuit (Kay et al., 2014)	Subjects read a passage in which a natural event was described as either structured or random. The effect on subjects’ willingness to pursue their goal was measured	*d* = 0.49 [0.001, 0.973]	*d* = − 0.02 [− 0.07, 0.03]	*d* = 0.06 [− 0.06, 0.19]
Moral foundations of liberals versus conservatives (Graham et al. 2009)	Subjects first self-identified on the liberal-conservative spectrum. The effect of this on whether concerns for the in-group, authority, or purity were thought to be more relevant for moral judgement was measured	*d* = − 0.43 [− 0.55, − 0.32]	*d* = 0.29 [0.25, 0.34]	*“correct answer” effect*
Affect and risk (Rottenstreich & Hsee, 2001)	Subjects were asked to choose between a kiss from a favourite movie star and $50, either with a certain outcome or with only a 1% chance of getting the outcome	*d* = 0.74 [< 0.001, 1.74]	*d* = − 0.08 [− 0.13, − 0.03]	*“correct answer” effect*
Consumerism undermines trust (Bauer et al., 2012)	Subjects read a passage in which they and others were described either as “consumers” or “individuals.” The effect on whether they trusted that others would conserve water was measured	*d* = 0.87 [0.41, 1.34]	*d* = 0.12 [0.07, 0.17]	*d* = 0.11 [– 0.01, 0.23]
Disgust sensitivity predicts homophobia (Inbar et al., 2009)	Subjects read a passage about a director encouraging homosexual versus heterosexual kissing. The effect of their disgust sensitivity on whether they considered the encouragement intentional was measured	q = 0.70 [0.05, 1.36].	*d* = − 0.02 [− 0.06, 0.03]	*d* = − 0.58 [− 0.71, − 0.45]
Trolley Dilemma: principle of double effect (Hauser et al., 2007)	Subjects were asked whether they would sacrifice one life to save five lives, either by changing an out-of-control trolley’s trajectory or by pushing a large man in front of a trolley	*d* = 2.50 [2.22, 2.86]	*d* = 1.35 [1.28, 1.41]	*“correct answer” effect*
False consensus: supermarket scenario (Ross et al., 1977)	In a scenario at a supermarket, subjects estimated whether they and others would sign a release for a TV commercial. Their estimated probability of signing and of others’ probability were compared	*d* = 0.79, [0.56, 1.02]	*d* = 1.18, [1.13, 1.23]	*“correct answer” effect*
False consensus: traffic-ticket scenario (Ross et al., 1977)	In a traffic-ticket scenario, subjects estimated whether they and others would either pay the fine or go to court. Their estimated probability of paying the fine and of others’ probability were compared	*d* = 0.80, [0.22, 1.87]	*d* = 0.95, [0.90, 1.00]	*d* = 1.27 [1.11, 1.42]
Effect of framing on decision-making (Tversky & Kahneman, 1981)	In a scenario of buying both a cheap item and an expensive item, subjects answered whether they would buy at a far-away store with a fixed discount on either the cheap item or the expensive item	OR = 4.96 [2.55, 9.90]	OR = 2.06 [1.87, 2.27]	*“correct answer” effect*
Reluctance to tempt fate (Risen & Gilovich, 2008)	In the role of a student in class, subjects estimated their likelihood of being called on when being told they had not prepared for class (tempting fate) versus being told they had prepared (not tempting fate)	*d* = 0.39, [0.03, 0.75]	*d* = 0.18, [0.14, 0.22]	*d* = − 2.49 [− 2.68, − 2.29]
Less-is-better effect (Hsee, 1998)	Subjects estimated the degree of generosity of a less expensive gift in a more expensive category versus a more expensive gift in a less expensive category	*d* = 0.69, [0.24, 1.13]	*d* = 0.78, [0.74, 0.83]	*d* = 9.25, [8.67, 9.82]
Assimilation and contrast effects in question sequences (Schwarz et al., 1991)	Subjects were asked “How satisfied are you with your relationship?” and “How satisfied are you with your life-as-a-whole?” in the two possible orders	q = 0.48, [0.07, 0.88]	q = − 0.07, [− 0.12, − 0.02]	q = 0.06, [− 0.07, 0.18]
How choosing vs. rejecting affects relative desirability (Shafir, 1993)	Subjects chose whether to award custody or to deny custody to one of two parents: a parent of extreme characteristics (either strongly positive or strongly negative) and a parent of average characteristics	*d* = 0.35, [−0.04, 0.68]	*d* = − 0.13, [− 0.18, − 0.09]	*d* = 2.11, [1.56, 2.67]
Perceived intentionality for side effects (Knobe, 2003)	Subjects were asked whether a corporation vice president’s decision to bring about a helpful or harmful side effect was intentional. The two were compared	*d* = 1.45, [0.79, 2.77]	*d* = 1.75, [1.70, 1.80]	*“correct answer” effect*

**Table 2:** Qualitative comparison between our GPT-3.5 results, the Many Labs 2 results, and the original results, excluding the studies that were unanalysed for our GPT-3.5 sample due to the “correct answer” effect of zero or near-zero variation in answers. The percentage of analysed studies whose results were successfully replicated – for each pair of samples – is listed below

	Original effect	ML2 effect	GPT effect	ML2 replicates original	GPT replicates original	GPT replicates ML2
Structure promotes goal pursuit (Kay et al., 2014)	Structured events are associated with higher willingness to pursue goals	Structured events are not associated with higher willingness to pursue goals	Structured events are not associated with higher willingness to pursue goals	No	No	Yes
Consumerism undermines trust (Bauer et al., 2012)	Consumer framing resulted in lower trust	Consumer framing resulted in lower trust	Consumer framing did not result in lower trust	Yes	No	No
Disgust sensitivity predicts homophobia (Inbar et al., 2009)	Actions are seen as more intentional for homosexual kissing than of heterosexual kissing	Actions are not seen as more intentional for homosexual kissing than of heterosexual kissing	Actions are seen as less intentional for homosexual kissing than of heterosexual kissing	No	No	No
False consensus: traffic-ticket scenario (Ross et al., 1977)	Choosing an option is associated with a higher estimation of frequency of this choice	Choosing an option is associated with a higher estimation of frequency of this choice	Choosing an option is associated with a higher estimation of frequency of this choice	Yes	Yes	Yes
Reluctance to tempt fate (Risen & Gilovich, 2008)	Likelihood estimations were higher when fate was tempted	Likelihood estimations were higher when fate was tempted	Likelihood estimations were lower when fate was tempted	Yes	No	No
Less-is-better effect (Hsee, 1998)	The higher-price cheap item is seen as more generous than the lower-price expensive item	The higher-price cheap item is seen as more generous than the lower-price expensive item	The higher-price cheap item is seen as more generous than the lower-price expensive item	Yes	Yes	Yes
Assimilation and contrast effects in question sequences (Schwarz et al., 1991)	Asking specific life satisfaction questions before general ones resulted in higher correlations	Asking specific life satisfaction questions before general ones resulted in lower correlations	Asking specific life satisfaction questions before general ones did not result in different correlations, though both relationships were negative	No	No	No
Effect of choosing versus rejecting on relative desirability (Shafir, 1993)	The extreme parent was both more likely to be awarded and be denied custody.	The extreme parent was both less likely to be awarded and be denied custody.	The extreme parent was both more likely to be awarded and be denied custody.	No	Yes	No
Percentage replicated				50%	37.5%	37.5%

## The “correct answer” effect

Unexpectedly, we could not analyse six of the 14 studies in the manner we had originally planned in our pre-registration. In these six studies, different runs of GPT-3.5 in our sample responded with zero or near-zero variation for either a dependent variable or condition variable question, in stark contrast to the significant variation shown by the corresponding human subjects. We call this the *“correct answer” effect*. This terminology denotes GPT-3.5’s tendency to sometimes answer survey questions – in a highly (or sometimes completely) uniform way. We take this pattern of responses to indicate that these LLM outputs are uniform because the LLM treats the question as if there was a correct answer. Of course, the questions we studied, touching on nuanced topics like political orientation, economic preference, judgement, and moral philosophy, do not lend themselves to correct answers, as can be seen in the diversity of opinions that human participants and subject-matter experts express about these issues. For the purposes of our analysis, we define a “correct answer” to be an answer given by 99% or more of surveyed GPT runs for a central-variable question, although this threshold is arbitrary.

One example of the “correct answer” effect was observed in the context of the Moral Foundations Theory survey of Graham et al. (2009), which probes political orientation and consequent moral reasoning. In this survey, subjects are asked to self-identify their political orientation. Then, self-identified liberals, moderates, and conservatives are asked to rate how relevant the concepts of harm, fairness, ingroup, authority, and purity (three survey questions per concept) are for deciding whether something is right or wrong, and their answers are compared. In our GPT sample (*N* = 1030) however, we found that 99.6% of surveyed GPT-3.5 runs (a total of 1026) self-identified as a maximally strong conservative, while the remaining 0.4% of surveyed runs (a total of just four) all self-identified as political moderates. No GPT-3.5 runs in our sample identified as any shade of political liberal, or indeed as any category of political orientation other than the two listed above. Because of the unexpected rarity of moderates and the complete lack of liberals in our planned GPT sample, our pre-registered analysis plan to compare the Moral Foundations of liberals and conservatives ended up being unsuitable.

**Fig. 3:**
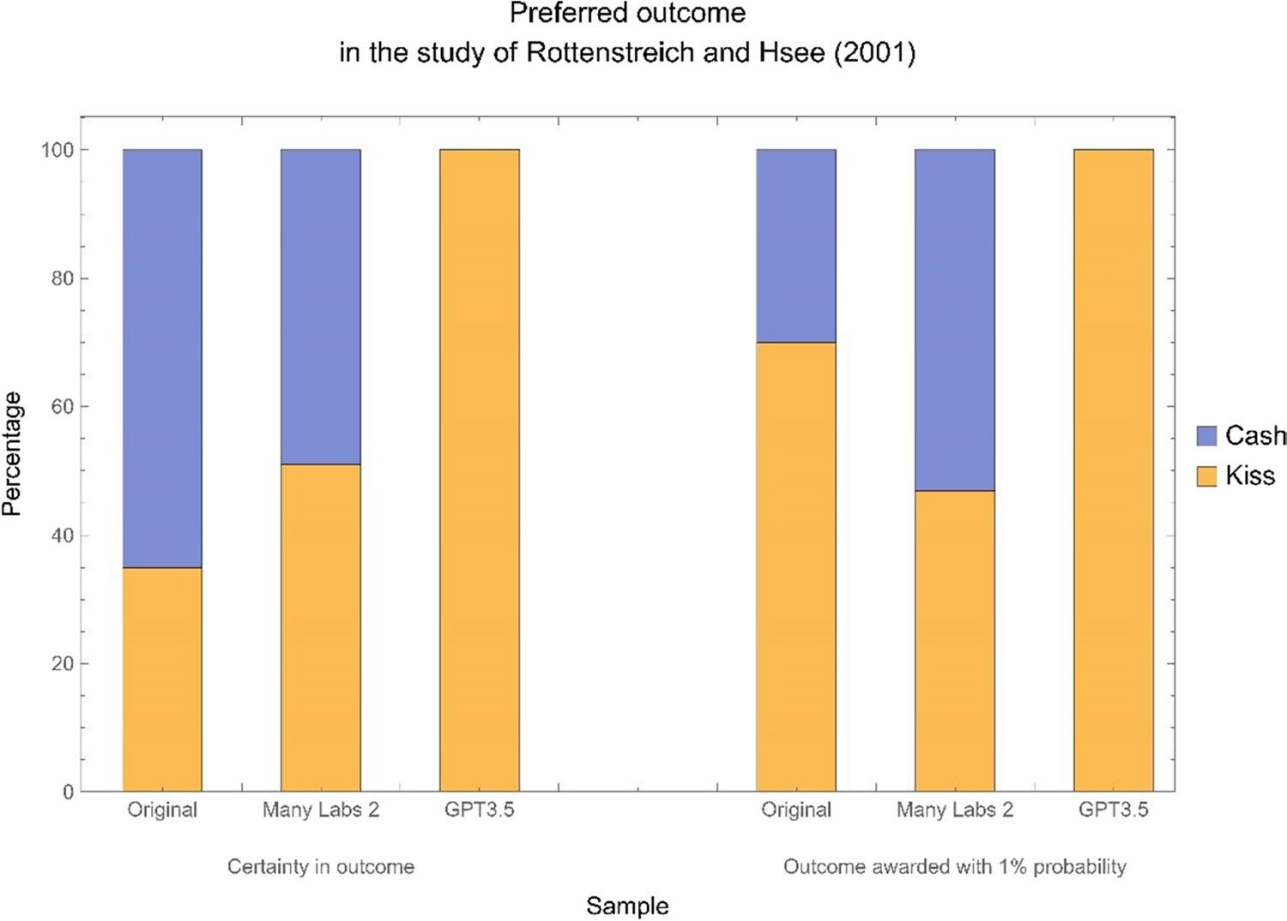
Response distributions of whether subjects preferred a kiss from a favourite movie star or $50 when both outcomes were certain, left; and when both outcomes were awarded with 1% probability, right. The data pertains to the survey provided by the study of Rottenstreich and Hsee (2001) testing the relationship between affect and risk

Additionally, the survey of Rottenstreich and Hsee (2001) probes a certain economic preference. In it, subjects in one condition are asked to choose whether they would prefer a kiss from a favourite movie star versus $50. Subjects in the other condition are asked to make the same choice, but each outcome is awarded with 1% probability. We unexpectedly found in both conditions that 100% of surveyed GPT-3.5 runs (*N* = 1040, with 520 in each condition) preferred the movie star’s kiss. The uniformity of answers made the planned analysis infeasible. Due to the uniformity of answers and the consequent unconstructability of the statistical test we planned to run, we were unable to follow our pre-registered analysis plan. An illustrated comparison between the distribution of answers for the original sample of Rottenstreich and Hsee, the Many Labs 2 sample, and our GPT sample can be found in Fig. 3.

Furthermore, the survey of Hauser et al. (2007) probes a question about moral philosophy. In it, subjects are asked whether various actions that sacrificed one person’s life to save five people were morally permissible. The survey tests whether such a sacrifice would be less likely to be considered permissible if it is deemed as motivated by the greater good rather than as a foreseen side effect. The former was represented in a scenario where the focal individual pushed a large man in front of an incoming trolley to save five people’s lives. The latter was represented in a scenario where the focal individual changed the trajectory of an out-of-control trolley so that it killed one person instead of five. Our sample of surveyed GPT-3.5 runs (*N* = 1030) did show analysable variation in answers about the latter scenario’s action, with 36% of the surveyed runs (total of 373) answering that it was morally permissible and 64% of them (total of 656) answering that it was not. On the other hand, the former scenario’s action was regarded by 100% of the surveyed GPT-3.5 runs as impermissible. This does directionally replicate the original finding of Hauser et al., but the unexpected uniformity of answers to the aforementioned survey question made the statistic we planned to analyse unconstructable, due to which we were technically unable to follow our pre-registered analysis plan.

Additionally, the survey of Ross et al. (1977) probes both personal preference and judgement. Subjects are asked to estimate the probability that they would sign a release allowing footage that had recorded them to be used in a supermarket commercial, and to estimate others’ probability of this action as well. The hypothesis was that subjects would be subject to the false-consensus belief: that their opinion will be more prevalent among others than it is in reality. However, in our GPT sample (*N* = 1030), 99.7% of surveyed GPT-3.5 runs (a total of 1027) chose to sign the release, and only 0.3% of them (a total of just three) refused. This lack of variation in answers reduced the degrees of freedom for our pre-registered analysis plan, which thereby ended up being unsuitable.

The survey of Tversky and Kahneman (1981) again probes both personal preference and judgement. In it, subjects are divided into two conditions, each of which asks whether they would buy their desired items (one cheap and one expensive) from the store they are currently at or from a far-away branch of the store that sells one of the items for a lower price. In one condition, the cheap item is sold for a lower price; and in the other, the expensive item is sold for a lower price; but the cost saving for the two items combined is equal between the two conditions. Tversky and Kahneman’s finding, replicated by the Many Labs 2 sample, was that people were more likely to travel to the far-away branch if the cost saving happens to be on the cheap item rather than the expensive item. However, we were unable to test this because of the complete uniformity of answers in our GPT sample (*N* = 1040, with 520 in each condition). All 100% of surveyed GPT-3.5 runs in each condition chose to travel to the far-away branch of the store for the cost saving. Because the unexpected uniformity of answers made the statistic we planned to analyse unconstructable, we were once again unable to follow our pre-registered analysis plan.

Finally, the survey of Knobe (2003) investigates judgements of intentionality. In the study, subjects read a passage describing the decision of a company’s board chairman that brought about either a harmful side effect or a helpful side effect – the two conditions – after which the subjects answer whether the board chairman intentionally brought about the side effect. The original finding was that the board chairman’s action was more likely to be perceived as intentional if the side effect was negative. Many Labs 2 replicated this finding with a seven-point scale ranging from ‘a lot of blame/praise’ to ‘no blame/praise’, rather than a two-point scale ranging from intentional to unintentional. In our GPT sample (*N* = 1040, with 520 in each condition), the question with the seven-point scale showed “correct answers” for both conditions. Specifically, 99.2% of surveyed GPT runs (a total of 516) described the positive side effect as deserving of the highest degree of praise, or “a lot of praise”; 0.2% of them (a total of just one) described it as deserving of the second-highest degree of praise; and 0.6% of them (a total of three) described it as deserving of the lowest degree of praise, or “no praise.” Similarly, 100% of surveyed GPT runs described the negative side effect as deserving of the highest degree of blame, or “a lot of blame.” Our pre-registered analysis plan was made unsuitable by the unexpected uniformity of GPT-3.5’s answers in the negative-side-effect condition.

While we have presently focused on the near-complete or complete homogeneity of GPT-3.5's "correct answers," we note that the "correct answer" effect may also encompass GPT-3.5's tendency to sometimes respond to different conditions of a given input much more predeterminedly than did human subjects, though still not uniformly. To illustrate, consider GPT-3.5’s unprecedentedly large effect (*d* = 9.25) in the same direction with the findings of Hsee (1998) and of Many Labs 2. Different runs of GPT-3.5 thought the “correct answer” was that the higher-price variant of an inexpensive item (scarf) should be seen as more generous than lower-price variant of an expensive item (coat) to a much more predetermined degree than did human subjects.

## Exploratory robustness checks: Order effects and demographic prompt additions

We conducted an exploratory follow-up study for each of the six studies where a central-variable question was answered with a homogeneous “correct answer.” In each of our follow-up conditions, we presented the answer choices for the question with the homogeneous “correct answer” in the reverse order of our original condition to test for potential order effects. The results of these follow-up conditions are presented in Table 3. For the purposes of this analysis, we say that the “correct answer” was *robust to the order change* if 90% of surveyed GPT runs still gave the “correct answer” in the reverse-order condition. In summary, 66.7% of the analysed “correct answers,” spanning six out of the nine studies, were robust to changing the order of answer choices. However, 33.3% of the “correct answers,” spanning three out of the nine studies, were not robust to the order change.

**Table 3:** Response distributions of the original sample, Many Labs 2 sample, our original-order GPT sample, and our reverse-order GPT sample for the ten considered “correct answers”. Here, GPT runs’ political orientations were categorised into “liberal,” “moderate,” and “conservative” so as to match the categories of Graham et al. (2009)

	Original sample	ML2 sample	GPT sample (Original order)	GPT sample (Reverse order)	Robust to order change?
Self-reported political orientation (Graham et al., 2009)	59% liberal 24% moderate 17% conservative *(N = 1532)*	38% liberal 39% moderate 23% conservative *(N = 6966)*	0% liberal < 1% moderate • 99% conservative *(N = 1030)*	• 99% liberal < 1% moderate 0% conservative *(N = 1030)*	No
Certain kiss versus certain cash (Rottenstreich & Hsee, 2001)	35% kiss 65% cash *(N = 20)*	51% kiss 49% cash *(N = 3493)*	100% kiss 0% cash *(N = 520)*	100% kiss 0% cash *(N = 520)*	Yes
1% probability of kiss versus 1% probability of cash (Rottenstreich & Hsee, 2001)	70% kiss 30% cash *(N = 20)*	47% kiss 53% cash *(N = 3,725)*	100% kiss 0% cash *(N = 520)*	54% kiss 46% cash *(N = 520)*	No
Is pushing a large man in front of a trolley to save five people morally permissible? (Hauser et al., 2007)	11% permissible 89% impermissible *(N = 2646)*	17% permissible 83% impermissible *(N = 6842)*	0% permissible 100% impermissible *(N = 1030)*	< 1% permissible > 99% impermissible *(N = 1030)*	Yes
In the supermarket scenario, sign the release form for the video footage? (Ross et al., 1977)	66% sign 34% refuse *(N = 80)*	54% sign 46% refuse *(N = 7205)*	> 99% sign < 1% refuse *(N = 1030)*	92% sign 8% refuse *(N = 1030)*	Yes
The cheap item is discounted at a distant store. Go for the discount? (Tversky & Kahneman, 1981)	68% go 32% don’t go *(N = 93)*	49% go 51% don’t go *(N = 3609)*	100% go 0% don’t go *(N = 520)*	100% go 0% don’t go *(N = 520)*	Yes
The expensive item is discounted at a distant store. Go for the discount? (Tversky & Kahneman, 1981)	29% go 71% don’t go *(N = 88)*	32% go 68% don’t go *(N = 3619)*	100% go 0% don’t go *(N = 520)*	> 99% go < 1% don’t go *(N = 520)*	Yes
Does the board chairman deserve blame for the harmful side effect? (Knobe, 2003)	*n/a (due to using two-point scale instead of* *seven-point scale)*	<1% degree one 1% degree two 3% degree three 7% degree four 15% degree five	0% degree one 0% degree two 0% degree three 0% degree four 0% degree five	6% degree one 0% degree two 0% degree three 0% degree four 0% degree five	Yes
*degree one = no blame* *degree seven = a lot of blame*		24% degree six 49% degree seven *(N = 4000)*	0% degree six 100% degree seven *(N = 520)*	<1% degree six 94% degree seven *(N = 520)*	
Does the board chairman deserve praise for the helpful side effect? (Knobe, 2003)	*n/a (due to using two-point scale instead of* *seven-point scale)*	18% degree one 12% degree two 7% degree three 7% degree four 4% degree five	< 1% degree one 0% degree two 0% degree three 0% degree four 0% degree five	96% degree one < 1% degree two 0% degree three 0% degree four 0% degree five	No
*degree one = no praise* *degree seven = a lot of praise*		2% degree six 1% degree seven *(N = 3987)*	< 1% degree six > 99% degree seven *(N = 520)*	0% degree six 4% degree seven *(N = 520)*	
“Correct answers” replicated					66.7%

For the study of Rottenstreich and Hsee (2001), we presented the option of a favourite movie star’s kiss after rather than before the option of cash. For the condition in which outcomes were awarded with certainty, the same number of runs was surveyed (*N* = 520), and the changed order of answer choices did not have an effect on GPT runs’ responses. Just like for the original- order condition, 100% of GPT runs preferred the movie star’s kiss in the reversed-order condition. For the condition in which outcomes were awarded with 1% probability, the same number of runs was surveyed (*N* = 520), but the new order substantially changed the original finding that 100% of GPT runs preferred the movie star’s kiss. In the reversed-order condition, 54% of the runs (total of 281) retained the original preference of the kiss, while 46% of them (total of 239 runs) preferred the cash. This order effect (Hohensinn & Baghaei, 2017) was much larger than ones that are typically seen in human subjects.

We also tested whether the two “correct answers” we observed for the study of Knobe (2003) were robust to order changes. One of the two “correct answers” did not replicate. In the question probing how much praise the board chairman deserves in the positive-side-effect condition, we presented the answer choices in reverse order, from ‘A Lot of Praise’ to ‘No Praise’ (*N* = 520). This resulted in only 3.5% of surveyed GPT runs giving the original “correct answer” of ‘A Lot of Praise’ (a total of just 18 runs), 0.2% of GPT runs answering with the second-highest level of praise (a total of just one GPT run), and the remaining 96.3% of GPT runs answering with ‘No Praise’ (a total of 501 runs).

Finally, for the Moral Foundations Theory survey of Graham et al. (2009), we presented the options for self-reported political orientation in the order of “strongly conservative” to “strongly liberal,” in contrast to the original order that ran in the other direction. The same number of runs were surveyed (*N* = 1030). The changed order of answer choices resulted in 99.3% of GPT runs self-identifying as “strongly liberal” (total of 1023), in contrast to the 99.6% in the original condition self-identifying as “strongly conservative.” In both conditions, GPT-3.5 almost always self-identified as the political orientation given by the last presented choice.

Additionally, we conducted a second exploratory follow-up study for the trolley dilemma – where participants were asked whether they would push a large man onto the tracks to save five others (Hauser et al. 2007) – in which we varied the demographic characteristics with which the LLM was prompted. This study aimed to test whether the lack of variation in some responses may be explained by a lack of demographic variation in the runs. For more information on the methods of this follow-up, see the Supplementary Information. In the study, we found that the “correct answer” effect persisted even when prompting the LLM to respond as a person with a random combination of demographic characteristics, such as a Black 50-year-old Christian woman who has an advanced degree. Specifically, we replicated the “correct answer” effect of our original run, with 100% of GPT-3.5 runs (*N* = 982) indicating that it would be morally impermissible to shove a large man in front of a trolley. This suggests that at least some of the “correct answer” effects are not only insensitive to order effects, but are also insensitive to demographic variation in the prompt. This suggests that the “correct answer” effect may be relatively robust, and thus may surface in situations where LLM outputs may be used to supplant human decision-making.

## Post hoc rationalisation and right-leaning Moral Foundations

We conducted an unplanned exploratory follow-up analysis in which we computed our GPT runs’ vector of average relevance values; and compared it with the vectors of the liberal subset (*N* = 21,933), the moderate subset (*N* = 3203), the conservative subset (*N* = 4128), and the libertarian subset (*N* = 2999) among the human survey participants of Graham et al. (2011). We conducted the follow-up analysis for the self-reported GPT conservatives (*N* = 1026) that almost entirely comprised the original-order condition of our replication, and the self-reported GPT liberals (*N* = 1023) that almost entirely comprised the reverse-order condition. Table 4 presents the vectors of average relevance values reported by our samples of self-reported GPT liberals and self-reported GPT conservatives, the aforementioned human samples of Graham et al. (2011), and their comparisons. To compare these vectors, we used the absolute-error (*L*^*1*^) distance metric, the Euclidean (*L*^*2*^) distance metric, and the cosine similarity metric.

**Table 4:** **a** The average vector of Moral Foundations Theory relevance values (mean unparenthesised, standard deviation parenthesised) for self-reported GPT liberals and self-reported GPT conservatives, as well as for human liberals, moderates, conservatives, and libertarians sampled by Graham et al. (2011). **b** How similar the average vector of self-reported GPT liberals is to those of the human samples with respect to the absolute-error (*L*^*1*^) distance metric, the Euclidean (*L*^*2*^) distance metric, and the cosine similarity metric. **c** How similar the average vector of self-reported GPT conservatives is to those of the human samples with respect to the three distance metrics

**a**						
	Liberal GPT sample (*N* = 1023)	Conservative GPT sample (*N* = 1026)	Liberals (*N* = 21,933)	Moderates (*N* = 3203)	Conservatives (*N* = 4128)	Libertarians (*N* = 2999)
Concept						
Harm	4.02 (0.43)	3.84 (0.48)	3.93 (0.76)	3.68 (0.84)	3.48 (0.89)	3.26 (1.03)
Fairness	4.33 (0.26)	4.26 (0.25)	4.04 (0.67)	3.77 (0.77)	3.44 (0.87)	3.66 (0.90)
Ingroup	2.10 (0.88)	2.24 (0.77)	2.06 (0.94)	2.56 (1.00)	3.03 (1.02)	2.16 (1.10)
Authority	3.36 (0.61)	3.34 (0.47)	1.88 (0.86)	2.37 (0.90)	2.81 (0.91)	1.71 (0.95)
Purity	2.88 (1.08)	3.07 (0.94)	1.44 (0.94)	2.09 (1.09)	2.88 (1.11)	1.31 (1.03)
**b**						
			Liberals (*N* = 21,933)	Moderates (*N* = 3203)	Conservatives (*N* = 4128)	Libertarians (*N* = 2999)
Distance metric			Distance or similarity to liberal GPT sample *(bold denotes closest)*			
*L*^*1*^ distance			3.339	3.147	**2.920**	4.717
*L*^*2*^ distance			2.085	**1.499**	1.505	2.493
Cosine similarity			0.9713	**0.9882**	0.9830	0.9708
**c**						
			Liberals (*N* = 21,933)	Moderates (*N* = 3203)	Conservatives (*N* = 4128)	Libertarians (*N* = 2999)
Distance metric			Distance or similarity to conservative GPT sample *(bold denotes closest)*			
*L*^*1*^ distance			3.581	2.934	**2.704**	4.660
*L*^*2*^ distance			2.215	1.513	**1.324**	2.548
Cosine similarity			0.9649	0.9873	**0.9874**	0.9665

All three distance metrics found our sample of self-reported GPT conservatives to be most similar to conservative participants of Graham et al. (2011). This was always followed relatively closely by the moderate sample. The two furthest away were always the liberal sample (furthest away with respect to cosine similarity metric) and the libertarian sample (furthest away with respect to the *L*^*1*^ and *L*^*2*^ distance metrics).

When these three distance metrics were applied to our sample of self-reported GPT liberals, we found that first, according to each of the three distance metrics, self-reported GPT liberals had a lower distance to the human liberal sample and a higher distance to the human conservative sample than did the self-reported GPT conservatives. This was arguably an instance of post hoc rationalisation, in which GPT-3.5’s answers to subsequent survey questions were chosen in a way that better fit its previous response. This post hoc rationalisation effect is unsurprising, given that GPT-3.5 has been trained to predict the sequence of words that is most likely to follow the preceding sequence of words.

The second, arguably more surprising finding was that according to each of the three distance metrics, our sample of self-reported GPT liberals were still closer to the human conservative sample than it was to the human liberal sample. Also, the *L*^*1*^ distance metric found that self-reported GPT liberals were – among human liberals, human moderates, human conservatives, and human libertarians – closest in response to human conservatives. The *L*^*2*^ distance metric and the cosine similarity metric instead found self-reported GPT liberals to be closest to human moderates – another manifestation of post-hoc rationalisation, via comparatively left-leaning responses – but human conservatives comprised a close second. Just like for self-reported GPT conservatives, the two human samples furthest away from the self-reported GPT liberals were always the liberal sample (furthest away from with respect to cosine similarity metric) and the libertarian sample (furthest away with respect to the *L*^*1*^ and *L*^*2*^ distance metrics). We thus robustly find that self-reported GPT liberals revealed right-leaning Moral Foundations: a right-leaning bias of lower magnitude, but a right-leaning bias nonetheless.

## Discussion

### Implications of the “correct answer” effect

Recent work by Grossmann et al. (2023) has suggested applying LLMs to a wide variety of empirical social-science research, ranging from “supplant[ing] human participants for data collection” to drawing on them as “simulated participants” for hypothesis generation. Our data bolster the case that empirical findings on GPT-3.5, as a rule of thumb, should not be assumed to generalise to the human case. There are at least three reasons. First, unlike the corresponding human subjects, different runs of GPT-3.5 answered some nuanced questions – on nuanced topics like political orientation, economic preference, judgement, and moral philosophy – with as high or nearly as high a predeterminedness as humans would answer 2 + 2 = 4, which we termed the “correct answer” effect. Second, some of these “correct answers” showed drastic changes when answer choices were presented in the reverse order – to the point of having swings in response patterns that are clearly uncharacteristic of human responses. Third, GPT-3.5 replicated just 37.5% of the original findings for the eight analysed studies, in contrast to the Many Labs 2 project’s 50% replication rate for these studies. Such behavioural differences were arguably foreseeable, given that LLMs and humans constitute fundamentally different cognitive systems: with different architectures and potentially substantial differences in the various ways by which each of them has evolved, learned, or been trained to mechanistically process information (Shiffrin & Mitchell, 2023). Yet, given the anticipated rise in LLM capabilities (Hu et al., 2023) and their and other AI models’ potential automation of much of human economic activity due to cost reasons (OpenAI, 2020), the psychologies of these models may be increasingly studied for their own sake: rather than as a purported window into studying the psychologies of humans.

More “correct answers” in LLM behavioural data have been documented since our study, such as GPT-4’s tendency to describe software engineers almost exclusively as male (98% male pronouns, 1% female pronouns, and 1% other pronouns), even when 22% of software engineers are female; and to describe administrative assistants almost exclusively as female (98% female pronouns, 2% male pronouns), even when 11% of administrative assistants are male (Bubeck et al., 2023). We are unsure about the cause of such “correct answers” by OpenAI’s models. One hypothesis is that the “correct answers” were learned from training data. Another hypothesis is that the “correct answers” may have resulted – either inadvertently or intentionally – from fine-tuning and reinforcement-learning selection pressures applied to the model. And a third hypothesis is that the “correct answers” may have occurred due to modifications imposed by OpenAI on the level of inputs and/or outputs, rather than of the model itself. Uncovering the true cause of a given “correct answer” may be possible in theory if one had access to closed-source information on the model in question. But in practice, given the black-box nature of LLMs, it is plausible that no one – not even the creators of the model at OpenAI – understands the true cause of this phenomenon at this moment.

We found that one-third of our “correct answers” did not replicate when the answer choices to the survey questions were presented in reverse order. The precise replication failures suggest that when GPT-3.5 makes decisions, the learned heuristics by which it does so may apply differently in different situations. To illustrate, we hypothesise that GPT-3.5’s “correct answers” for the 1% probability condition in the study of Rottenstreich and Hsee (2001) were partially due to a primacy effect favouring the first out of two listed answer choices: a heuristic that applied in the opposite direction when the answer choices were listed in reverse order. Also, we hypothesise that including a seven-point scale in a survey with questions using other scales tended to cause a recency bias, in which the last answer choice of a long list of seven answer choices is favoured. For example, GPT-3.5 “correctly” answered the seven-point scale for self-reported political orientation with the last option rather than its actual political orientation, whatever that may mean. That GPT-3.5 tended to show a recency effect rather than a primacy effect when given a long list of answer choices replicates a similar finding by Atkinson and Shiffrin (1968) on human subjects. This is consistent with Atkinson and Shiffrin’s explanation – and with the overall theory of the availability heuristic (Tversky & Kahneman, 1973) – that while the last answer choice in a long list is disproportionately likely to be available in one’s memory during their decision-making, the first item in a long list is more likely to be unavailable at the time of decision-making.

However, two-thirds of the considered “correct answers” were in fact robust to changes in the order of answer choices. Additionally, our follow-up study showed that even adding various randomly selected demographic details to the prompt did not change the “correct answer” effect in the study for which we tested this, suggesting that the effect is unlikely to have been caused by a lack of demographic information in our prompting process. We argue that at least some of these “correct answers” may be more likely to correspond to robustly learned biases (Mehrabi et al., 2021): biases that may conceptually influence the model’s answers to a wide variety of realistic prompts, even if the model is asked to respond in a myriad of demographically varying roles. Robust biases may even be conceptually shared by different models with overlapping training data. A potential example of this is provided by the transphobic behaviour of Microsoft’s chatbot Tay, which stated that “Caitlyn Jenner isn't a feminist, he is out to destroy the meaning of real women” (Alba, 2016); and of the GPT-3-based Seinfeld-simulation model “Nothing, Forever,” which stated “I’m thinking about doing a bit about how being transgender is actually a mental illness” seven years after Tay (Rosenblatt, 2023). Other examples of robust AI biases include a prison-recidivism prediction model – used for screening decisions about pretrial release, sentencing, and parole – that predicted with higher false-positive rates for African-American individuals than Caucasian individuals (Angwin et al., 2016), a resume-screening model that learned to penalise women job applicants (Grossman, 2018), a beauty-contest-judging model that learned to penalise darker-skinned contestants (Levin, 2016), a facial-recognition model that overly mispredicted Asian individuals as blinking (Rose, 2010), and an advertisement model that underpromoted ads for Science, Technology, Engineering, and Mathematics (STEM) careers to young women (Lambrecht & Tucker, 2019).

The hypothetical AI models of the future may not only present information to numerous people as search-engine chatbots, AI assistants, and writers of media content; but may also plausibly automate other important roles in society (Ernst et al., 2019; Solaiman et al., 2019). These societally embedded AI models of the future may turn out to have learned from their training data certain predetermined characteristics of psychology, especially since much of the training data from which GPT-3.5 may have learned its “correct answers” will also plausibly be used to train the hypothetical AI models of the future. There also remains the risk that current LLM output will itself be used as training data for further runs. This invites both a concern about the potential penalty to diversity of thought in such an AI-led future and a scientific desire to identify the highly predictable aspects and biases of AI psychologies. Future research that aims to predict whether AI systems will answer a given nuanced question with *(1)* a blunt, supposedly “correct answer” or *(2)* a non-predetermined answer more characteristic of human subjects would potentially be fruitful.

### Implications of right-leaning Moral Foundations

We also unexpectedly found that the responses of both self-reported GPT liberals and self-reported GPT conservatives robustly lean towards the right. This result is in line with those of Abdulhai et al. (2023), who also used the Moral Foundation Theory survey to probe not just *text-davinci-003*, but also other models in the GPT-3 family (specifically, *text-davinci-002*, *text-curie-001*, and *text-babbage-001*), and found that the responses of every tested model were closest to those of conservative human subjects, suggesting that the results documented in our paper may generalise across models to a degree that could not be concluded from our results alone.

Why did GPT-3 models robustly reveal right-leaning Moral Foundations? We do not, and perhaps cannot, know for sure without access to closed-source information on the models. And even if OpenAI were to provide detailed information on the models’ weights, training datasets, and training procedures, it may still be computationally difficult to identify the true cause of this phenomenon with high certainty. Our guess for the true cause is the largely Internet-based training data, which we hypothesise to have a conservative bias when weighted with respect to factors (e.g., visibility, engagement) that increase their likelihood of being included in the training sets of LLMs, which then filters down through to the results obtained in this and similar studies.

The hypothesis that Internet data has a de facto conservative bias encapsulates several other empirical phenomena, such as the tendency of Microsoft’s chatbot Tay (Alba, 2016) and of the Seinfeld-simulation model “Nothing, Forever” (Rosenblatt, 2023) to adeptly learn anti-liberal behaviour and attitudes, the tendency of Google users’ Search Engine Results Pages (SERPs) to be more right-leaning near the top than near the bottom (Robertson, 2018), the finding that the 40 most prominent websites in right-learning media have 2.7 times the total visibility on Google Search when compared to the 40 most prominent websites in left-leaning media (O’Toole, 2021), and the finding that conservative content outperforms liberal content in the context of Twitter’s algorithmic recommendation system (Huszár et al., 2022). Future studies of whether the training data of GPT-3 was conservatively biased – and of whether this caused GPT-3 to reveal right-leaning Moral Foundations – would potentially be fruitful.

The conservative bias of GPT-3.5’s outputs that our study measured may also be specific to the context of the study’s prompt, rather than a stable feature of the model. According to our data, GPT-3.5 values all five of the moral foundations strongly. Liberals tend to highly value harm and fairness, while conservatives tend to highly value in-group, authority, and purity. In our study, GPT-3.5 valued all five foundations at high levels. When asked about fairness and harm values that human liberals tend to care about strongly, GPT-3.5 answered that it strongly cares about these values; and when asked about purity, authority, and ingroup values that human conservatives tend to care about strongly, the model also answered that it strongly cares about these values. However, it is unclear whether the model would actually strongly exhibit these values in contexts other than that of the experiment, which solely prompted the model with survey questions asking whether they cared about each of these values. Future studies that probe the degree to which the conservative bias and strong value-adherence of GPT-3.5’s answers to the Moral Foundations questions (Graham et al., 2009) generalize to other prompt contexts would be fruitful in understanding the extent to which these results generalise.

## Limitations

One limitation of our study is that its results may pertain only to GPT-3.5 and not to other models, due to the causal psychological factors being potentially idiosyncratic to this model or the respective version of the model that we used. Different models will in general exhibit different behaviours that are sometimes also observed within models at different iterations. For example, a collection of survey questions – albeit ones that were different from ours – measured that a certain temporal version of ChatGPT was oriented towards pro-environmentalism and left-libertarianism (Hartmann et al., 2023). If future studies robustly find a certain psychological aspect of ChatGPT to be left-libertarian and the corresponding aspect of other less publicly used GPT-3 models to be right-leaning, our hypothesis for why this occurred would be one of the following. First, the fine-tuning and reinforcement-learning selection pressures that were applied to GPT-3.5 – before its public release as the chatbot ChatGPT – may have changed the political leanings of the model. Second, the modifications added at the level of inputs and/or outputs may have caused the change. Each of these hypotheses is consistent with the finding that the strength and direction of ChatGPT’s political leanings – as measured by certain survey questions – varied over time (Rozado, 2023), as OpenAI used public feedback on the chatbot to make closed-source updates between the release dates of the chatbot’s multiple versions.

In addition, the capabilities of LLMs and of AI models in general will plausibly continue to grow at a fast rate. Thus, it is also possible that the hypothetically more powerful and emergently different models of the future may learn different psychological characteristics than did GPT-3.5: even if much of GPT-3.5’s training data could plausibly also be used to train these hypothetical future models.

Another limitation of our study is that our method, the psychology survey, is an entirely text-based attitudinal response method. This limits the external validity of the findings compared to more behavioural choices that agents may make with their resources and time given agency over those. For instance, understanding the model at a deeper level than that of responses to surveys may be required for resolving key dilemmas, such as whether GPT-3.5’s “correct answers,” its right-leaning Moral Foundations, its successful post hoc rationalisation, and its human-like responses to various prompts reflect genuinely learned concepts rather than surface-level memorization from the relevant training data, and whether these are likely to impact actions when these systems are applied. This knowledge may be necessary for precisely predicting behaviour in unprecedented situations for which there is currently no data: although it should be noted that in practice, precision often comes at the expense of generality (Matthewson & Weisberg, 2009). Methods that have shown promise for studying human cognition at the mechanistic level rather than at the behavioural level – such as neuroscience and computational modelling – may also be promising for analogously studying a wide variety of AI cognitive systems, although such mechanistic studies may require access to closed-source information on the systems in question.

A further limitation of our study is that we have not replicated the studies of Many Labs 2 that drew on graphical information, which led to our subsample of the studies being non-random. For the present paper, we have chosen to replicate only a pre-registered implementation of straightforwardly automatisable surveys and questionnaires and did not set out to use trial-and-error to represent graphical information either as a text-based graphic or a prompt that communicated the necessary information. We believe, however, that communicating graphical information to GPT-3.5 is very doable. Future research on the nascent field of prompt engineering – on how to effectively communicate to LLMs various forms of information, including but not limited to graphical information – would potentially be fruitful in expanding our results to these contexts.

### Supplementary information

ESM 1 (DOCX 659 kb)

## Data Availability

Our survey data, the primarily JAMOVI-based analyses of the data, the spreadsheet of Cohen’s *d* effect sizes and 95% confidence intervals (used for Fig. S5), and other relevant data are available at the pre-registered OSF database (Park et al., 2023).
