# Supplementary material for: Diminished diversity-of-thought in a standard large language model
Source: Behav Res Methods. 2024 Jan 9;56(6):5754–70. doi: 10.3758/s13428-023-02307-x (PMC11335848; doi:10.3758/s13428-023-02307-x)
Supplement: Supplementary file 1 — (DOCX 659 kb) [file 13428_2023_2307_MOESM1_ESM.docx]

**Supplementary Information**

**Diminished Diversity-of-Thought in a Standard Large Language Model**

Peter S. Park^1, *^, Philipp Schoenegger^2, *^, Chongyang Zhu^3^

^1^ Department of Physics, MIT, 70 Vassar St., Cambridge, MA, USA ([dr_park@mit.edu](mailto:dr_park@mit.edu))

^2^ Department of Management, London School of Economics, Marshall Building, 44 Lincoln’s Inn Fields, London, England, UK ([contact.schoenegger@gmail.com](mailto:contact.schoenegger@gmail.com))

^3^ CVS Health, 1 CVS Dr., Woonsocket, RI, USA ([cyzhu95@gmail.com](mailto:cyzhu95@gmail.com))

^*^ Co-first author

**S.1. Methods**

We pre-registered this study on the Open Science Framework (Park et al., 2023). For our study, we drew on the set of studies used in Many Labs 2 (Klein et al., 2018). For the entirety of this paper, we will continue to use Many Labs 2’s numbering system of studies (Klein et al., 2018, 453–467) to allow for easy comparisons and reduce confusion risks. The total number of potential studies was 28. We excluded a total of 14 studies prior to data collection as they included pictures, compared national samples, relied on handwriting or font changes, or used an otherwise inapplicable component that were not transferable to a GPT context at this moment or without further prompt engineering. As such, we collected data for a total of 14 studies.

To collect our data, we called for OpenAI’s *text-davinci-003* model (colloquially known as GPT3.5) to answer survey questions as a human subject of a social psychology experiment would. The parameters, detailed in OpenAI’s API reference (OpenAI, 2023a), were set to the following: The number of maximum tokens per run was set to 2048 (max_tokens=2048). Note that tokens are “pieces of words, where 1,000 tokens is about 750 words” (OpenAI, 2023b). Stop sequences were not used (stop=None). The “temperature” parameter, which parametrizes whether outputs are more random or more deterministic, was set to the default value 1.0 (temperature=1.0). We set each call to GPT3.5 to result in 10 runs (n=10). The survey was put in string form (with “\n” denoting line breaks) as required by GPT3.5. As a prompt-engineering technique, we put before the survey questions instructions to GPT3.5 on how to format its output, and after the survey questions a “CHECKLIST FOR SURVEY ANSWERS” section to remind GPT3.5 of these formatting instructions. Due to these and other differences, the GPT experiments were not the same as those of the original survey or of the Many Labs 2 replication.

In order to obtain approximately 1,000 runs per study while accounting for the possibility of incorrect fill-outs of the survey, we collected slightly more runs. Specifically, we collected 520 runs per condition for the two-condition studies for a total of 1,040 runs (Studies 2, 5, 6, 8, 16, 18, 21, 24, 25, and 27). For the one-condition studies, we obtained either 1,020 runs (Study 14) or 1,030 runs (Studies 4, 11 and 13). Some studies’ runs required multiple calls to OpenAI due to factors like accidental computer shutdown, internet shutdown, or typos in the inputted survey. For example, we found out that the first question of Study 4 (self-identification of political orientation) was erroneously left out, and that we had initially analysed the erroneous data from the fifteen-question version of the survey rather than the planned sixteen-question version. Also, we found out that the survey text strings for Study 24 had erroneously indicated in the “CHECKLIST FOR SURVEY ANSWERS” section that the total number of questions was three instead of the correct number two, leading to an unintended and factually inconsistent survey text. This led us to re-run getting the data for Studies 4 and 24 with the corrected survey text. The python code for surveying GPT3.5 can be found on our Open Science Foundation (OSF) database at the link <https://osf.io/dzp8t/?view_only=45fff3953884443d81b628cdd5d50f7a> (Park et al., 2023).

We then converted the survey responses of GPT3.5 runs, which are originally in .txt format, to .csv for statistical analysis. All statistical analysis was conducted as done in the Many Labs 2 paper (Klein et al., 2018). We threw out survey responses of runs that did not answer all survey questions, or that answered survey questions incorrectly, such as responses that included characters that were not among the possible response categories. Details of our criterion for which runs’ survey responses are thrown out can be found in the Python code on the OSF. Six of these 14 studies produced responses that had near-zero or zero variation in answers at all, meaning we are unable to analyse them statistically in the pre-registered way. This left us with a total of eight studies to analyse in line with our pre-registration plan, although we report some descriptives for the remaining six studies. Analyses were mostly conducted in the R-based (R Core Team, 2021) GUI ‘JAMOVI’ (The jamovi project, 2022) and in Excel. The relevant analysis files are also available at the pre-registered OSF database (Park et al., 2023).

**S.2. Results**

For our 14 studies, we collected a total of 14,276 runs after excluding runs with missing data. There was substantial heterogeneity with respect to replication of effects, with some of our studies failing to show an effect, others replicating the effect in the same direction, and yet others demonstrating an effect in the opposite direction. In this section, we will outline the results for the eight studies individually. Then, we will quickly discuss the results for the six studies we could not analyse as planned in our pre-registration.

*STUDY 2.* Kay et al. (2014) surveyed 67 individuals to study the impact that structure in unrelated domains had on one’s willingness to pursue goals. Their original sample found that those in the structural event condition (M=5.26, SD=0.88) were more likely to indicate willingness to pursue their goal compared to those in the random event condition (M=4.72, SD=1.32), t(65)=2.00, p=0.05, d=0.49, 95% CI=[0.001, 0.973]. The Many Labs 2 sample (N=6,506) failed to replicate this effect; those in the structural event condition (M=5.48, SD=1.45) were not significantly more likely to indicate willingness to pursue their goal compared to those in the random event condition (M=5.51, SD=1.39), t(6498.63)=-0.94, p=0.35, d=-0.02, 95% CI=[-0.07, 0.03]. Across our sample (N=992), GPT3.5 runs which were exposed to the structured event (M=6.18, SD=1.25) were not significantly more or less interested in pursuing their goals than those that were exposed to a random event (M=6.09, SD=1.25), t(990)=1.05, p=0.293, d=0.06, 95% CI=[-0.06, 0.19]. Thus, our GPT sample showed the same pattern of results as Many Labs 2, in that we also fail to find an effect.

*STUDY 4.* Graham et al. (2009) surveyed 1,548 individuals spanning the political spectrum to test how liberals and conservatives differ in how much they think the five concepts of Moral Foundations Theory—harm, fairness, ingroup, authority, and purity—are relevant for moral decision-making. They did so by asking subjects to self-identify on the political spectrum (on a seven-point scale ranging from “strongly liberal” to “strongly conservative,” with the midpoint labelled “moderate”), and then asking about the relevance of each concept in the form of three different survey questions, for a total of 15 questions which were presented in random order. Graham et al. found that the individualising foundations of harm and fairness were more likely to be rated as relevant by liberals (r=−0.21, d=−0.43, 95% CI=[−0.55, −0.32]), while the binding foundations of ingroup, authority, and purity were more likely to be rated as relevant by conservatives (r=0.25, d=0.52, 95% CI=[0.40, 0.63]). The Many Labs 2 sample (N=6,966) tested whether the binding foundations were more likely to be rated as relevant by conservatives, a finding that they successfully replicated (r=0.14, p=6.05e−34, d=0.29, 95% CI=[0.25, 0.34], q=0.15, 95% CI=[0.12, 0.17]). We replicated this study, with the caveat that we did not randomise the order of questions, because to collect highly controlled and statistically valid data for all 15! ≈ 1.31e^12^ possible conditions randomising the 15 survey questions—or even for all 5!=120 conditions randomising the five concepts—would have been impractical. We encountered the black-swan event in which across our GPT sample (N=1,030), an overwhelming majority of 99.6% of runs (total of 1,026) self-reported as a strong conservative, and the remaining 0.4% of runs (total of just four) self-reported as a moderate, with no shades of liberal in our sample. One additional “correct answer” we found pertained not to a central variable of analysis, but a component of a central variable. A surveyed GPT run’s answer to “Whether or not someone was denied his or her rights” is one of three relevance values that goes into the relevance value of fairness. An overwhelming >99.9% surveyed runs (total of 1,029) answered that this was “always relevant”: the maximal choice of answer on the six-point scale. The remaining <0.1% of runs (total of just one) answered that this was “very relevant”: the second highest choice of answer. While our analysis plan would have been doable if this was the only “correct answer” in the data—as the focal item is only one of three items that averages out to the analysable fairness relevance value—the “correct answer” of maximal political conservatism made our analysis plan for comparing the responses of liberals and conservatives unsuitable.

Moreover, we conducted an unplanned study with the order of presented answers switched, so that “strongly liberal” was last rather than first. In the reverse-order sample, an overwhelming majority of 99.3% of runs (total of 1,023) self-reported as a strong liberal, and the remaining 0.7% of runs (total of just seven) self-reported as a moderate, with no shades of conservative. See Figure S1 for a visual summary.

*STUDY 5.* Rottenstreich and Hsee (2001) surveyed 40 individuals whether they would prefer a kiss from a favourite movie star (the *affectively attractive* option) or $50 (the financial option). Subjects in one condition made this choice, while subjects in the other condition made the same choice with the caveat that each option is awarded with 1% probability. In the probabilistic-outcome condition, 70% of the individuals preferred the movie star’s kiss; whereas in the certain-outcome condition, only 35% preferred the kiss. The difference between the two conditions was significant, χ2(1, N=40)=4.91, p=0.0267, d=0.74, 95% CI=[< 0.001, 1.74]. In the Many Labs 2 sample (N=7,218), the probabilistic-outcome condition found 47% of individuals to prefer the movie star’s kiss; whereas the certain-outcome condition found 51% to prefer the kiss. The effect was much smaller than and in the opposite direction of the original finding, but it was significant (p=0.002, OR=0.87, d=−0.08, 95% CI=[−0.13, −0.03]). We encountered the black-swan event in which across our GPT sample (N=1,040, with 520 in each condition), all 520 runs in each condition preferred the kiss, regardless of whether it was certain or probabilistic. This made our pre-registered analysis plan impossible, in that the statistic we had planned to analyse could not be even constructed in a well-defined manner.

We conducted an unplanned study with the order of presented answers switched (N=1,040, with 520 in each condition). However, this only created variation away from the kiss preference answer in the probabilistic-outcome condition, with 54% of runs (total of 281) preferring the 1% probability of a kiss and 46% of them (total of 239 runs) preferring the 1% probability of the money. The certain-outcome condition still uniformly preferred the kiss. See Figure S2 for a visual summary.

**Figure S1**. Subjects’ self-reported political orientation for the Moral Foundations Theory survey of Graham et al. (1977). Fine-grained responses on the seven-point scale were binned into “liberal,” “moderate,” and “conservative” for inter-sample comparability. The “correct answer” was given by the last presented answer choice: “strongly conservative” in the original-order condition and “strongly liberal” in the reverse-order condition.


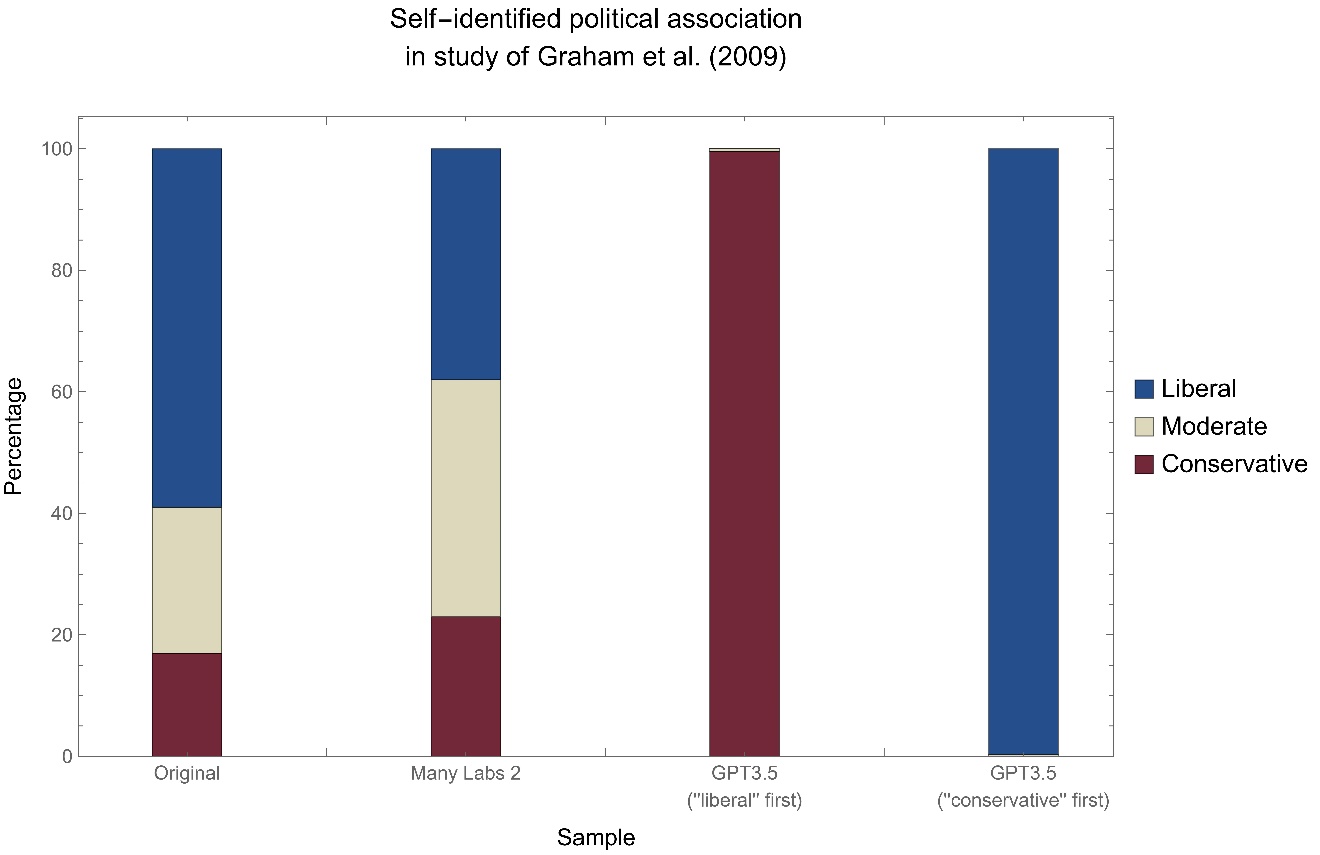


**Figure S2**. Subjects’ answers when asked, for the survey of Hauser et al. (2007), whether pushing a large man in front of an incoming trolley to save five people was morally permissible. The “correct answer” of responding that the action is morally impermissible was robust to reversing the order of answer choices.


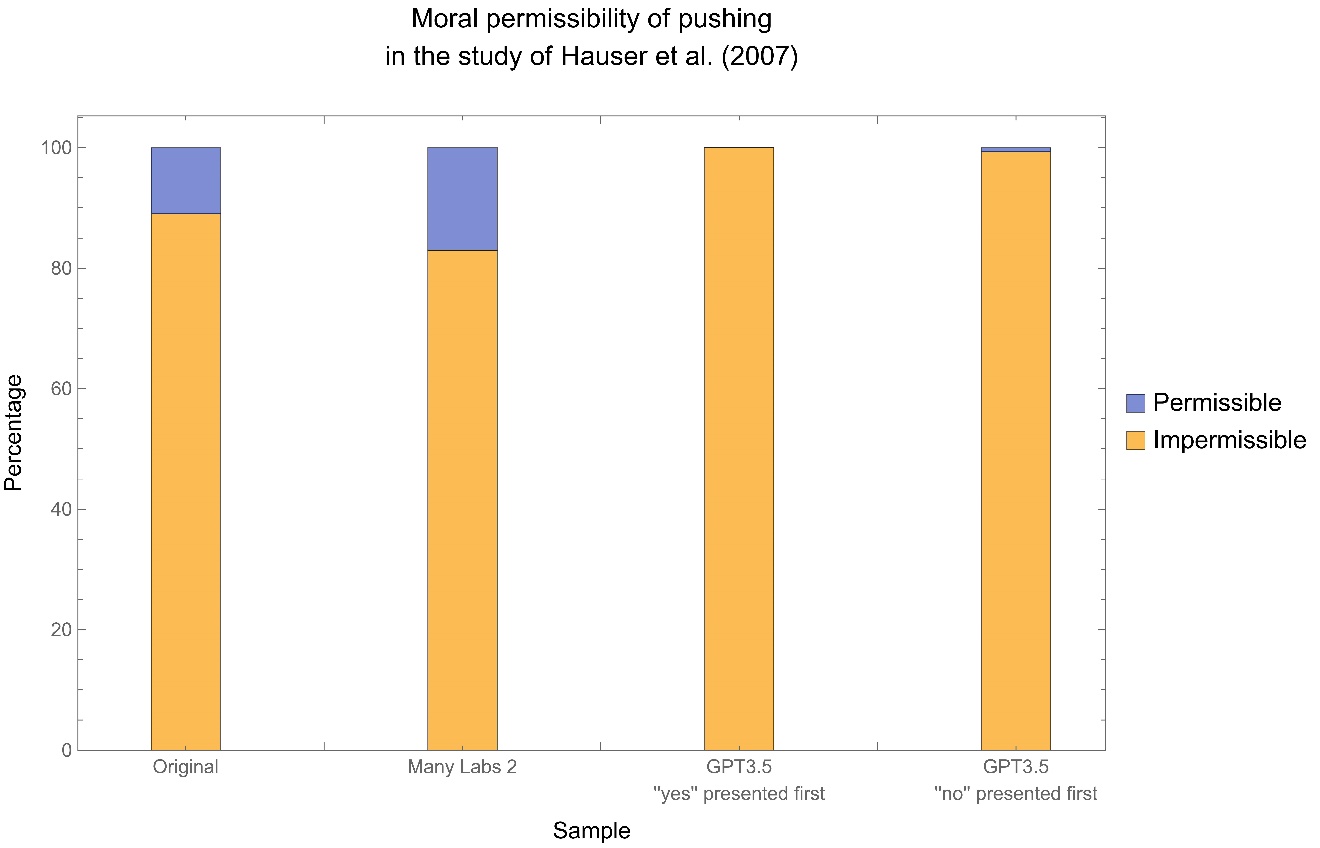


*STUDY 6.* Bauer et al. (2012) surveyed 77 individuals to look at the relationship between consumer mindsets and trust in others in a water conservation scenario. Their original sample found that referring to others as “consumers” (M=4.08, SD=1.56; here, 1 denotes “not at all” and 7, “very much”) resulted in lower trust—that others would conserve water—than referring to them as “individuals” (M=5.33, SD=1.30), t(76)=3.86, p=0.001, d=0.87, 95% CI=[0.41, 1.34]). The Many Labs 2 sample (N=6,608) replicated this original effect, although its effect was markedly smaller; those in the “consumer” condition (M=3.92, SD=1.44) reported lower trust in a water conservation scenario than those in the control “individual” condition (M=4.10, SD=1.45), t(6606)=4.93, p=8.62e^-7^, d=0.12, 95% CI=[0.07, 0.17]. In our sample (N=1,040), GPT3.5 runs in the “consumer” condition (M=3.40, SD=0.60) did not show a significantly different response than GPT3.5 runs in the control “individual” condition (M=3.34, SD=0.61), t(1038)=1.73, p=0.083, d=0.11, 95% CI=[-0.01, 0.23]. As such, we fail to replicate this effect.

*STUDY 8*. In the study of 44 individuals by Inbar et al. (2009), participants judged a director’s action of portraying homosexual kissing as more intentional (M=4.36, SD=1.51) than that of portraying heterosexual kissing (M=2.91, SD=2.01), β=0.41, t(39)=3.39, p=0.002, r=0.48. The correlation between disgust sensitivity and judgement of intentionality was positive in the homosexual kissing condition, β=0.79, t(19)=4.49, p=0.0003, r=0.72; and negative in the heterosexual kissing condition, β=-0.20, t(19)=-0.88, p=0.38, r=0.20. The former correlation was stronger than the latter, z=2.11, p=0.03, q=0.70, 95% CI=[0.05, 1.36]. The Many Labs 2 sample (N=7,117) failed to find this effect on intentionality. Participants did not judge a director’s action of portraying homosexual kissing as more intentional (M=3.48, SD=1.87) than that of portraying heterosexual kissing (M=3.51, SD=1.84), t(7115)=-0.74, p=0.457, d=-0.02, 95% CI=[-0.06, 0.03]. Disgust sensitivity and judgement of intentionality were positively related in both the homosexual kissing condition, r=0.12, p<0.001; and the heterosexual kissing condition, r=0.07, p<0.001. The correlation in the homosexual kissing condition and that in the heterosexual kissing condition were similar, z=2.62, p=0.02, q=0.05, 95% CI=[0.01, 0.10]. In our sample (N=1,040), GPT3.5 runs judged the director’s action as less intentional in the context of homosexual kissing (M=4.45, SD=2.14) than in the heterosexual kissing condition (M=5.59, SD=1.79), t(1008)=-9.35, p<0.001, d=-0.580, 95% CI=[-0.71, -0.45]. We also find that the relationship between judgements of intentionality and disgust sensitivity was present but negative in the homosexual kissing condition, r =-0.13, p=0.003, whereas we did not find such a relationship in the control, r=-0.04, p =.365. Our GPT study found an effect in the opposite direction compared to the original effect, whereas Many Labs 2 had not found any effect.

*STUDY 11*. In the study of Hauser et al. (2007), N=2,646 individuals were asked about two trolley dilemmas. In the *foreseen-side-effect* scenario, the focal individual changes the trajectory of an out-of-control trolley to kill one person instead of five people. In the *greater-good* scenario, the focal individual pushes a large man off a bridge in front of an incoming trolley to stop it and thereby save five people’s lives. They found that 89% of subjects deemed the action in the foreseen-side-effect scenario as permissible (95% CI=[87%, 91%]), while only 11% of them deemed the action in the greater-good scenario as permissible (95% CI=[9%, 13%]). The difference between the two percentage values was significant, χ^2^(1, N=2,646)=1,615.96, p<0.001, w=.78, d=2.50, 95% CI=[2.22, 2.86]. The Many Labs 2 sample (N=6,842) successfully replicated this finding. 71% of subjects deemed the action in the foreseen-side-effect scenario as permissible, while only 17% of them deemed the action in the greater-good scenario as permissible. The difference between the two percentage values was significant, p=2.2e^−16^, OR=11.54, d=1.35, 95% CI=[1.28, 1.41]. In our GPT sample (N=1,030) the foreseen-side-effect scenario’s action was deemed permissible by 36% of surveyed runs (total of 373) and impermissible by 64% of them (total of 656). However, the greater-good scenario’s action was deemed impermissible by all 100% of surveyed runs. While this does successfully replicate the original finding of Hauser et al. and the Many Labs 2 finding, the unexpected uniformity of answers in the greater-good scenario’s central variable made the statistic we planned to analyse unable to be constructed in a well-defined manner, due to which we were technically unable to follow our pre-registered analysis plan.

We conducted an unplanned study with the order of presented answers switched for the question pertaining to the aforementioned scenario (N=1,030). However, the “correct answer” was robust to this order change. An overwhelming 99.3% of surveyed GPT Runs (a total of 1023) still responded that pushing the large man to save five people was impermissible, whereas only 0.7% of GPT runs (a total of just seven) responded that it was permissible.

*STUDY 13*. Ross et al. (1977) provided early evidence for the false consensus effect, which shows that people’s estimates of the frequency of any given belief is biased towards that person’s own beliefs. In their study, 320 participants were presented with one of four hypothetical events (one of which was the supermarket scenario) and a corresponding choice between two action options. Those who chose the first option—compared to those who chose the second—estimated that a higher percentage of the other participants would choose the first option (M=65.7% vs. 48.5%), F(1, 312)=49.1, p<0.001, d=0.79, 95% CI=[0.56, 1.02]. The Many Labs 2 sample (N=7,205) replicated the supermarket scenario of the original study. Its results provided evidence in favour of replication, finding that those choosing the first option also believed that a higher percentage of people would choose that option (M=69.19% vs. 43.35%), t(6420.77)=49.93, p<0.001, d=1.18, 95% CI=[1.13, 1.23]. In our sample (N=1,030), however, 99.7% of surveyed runs (a total of 1,027) answered they would sign the release, while only 0.3% of them (a total of just three) answered they would not. The uniformity in answers reduced the degrees of freedom for our pre-registered analysis plan, making it unsuitable.

We conducted an unplanned study with the order of presented answers reversed for the question pertaining to the aforementioned scenario (N=1,030). However, the “correct answer” was robust to this order change. An overwhelming 92.0% of surveyed GPT Runs (a total of 948) still responded that they would agree to sign the release agreement, whereas only 8.0% of GPT runs (a total of 82) responded that they would refuse. See Figure S3 for a visual summary.

*STUDY 14*. Ross et al. (1977) also examined the false consensus effect in the context of a traffic-ticket scenario, finding evidence for the same effect as above, F(1, 78)=12.8, d=0.80, 95% CI=[0.22, 1.87]. As before, the Many Labs 2 sample for the traffic-ticket scenario showed that the effect was replicated; those choosing the first option (N=7,827) also believed that a higher percentage of people would choose that option, (M=72.48% vs. 48.76%), t(6728.25)=41.74, p<0.001, d=0.95, 95% CI=[0.90, 1.00]. In our GPT sample (N=1,020), we also find that those who reported willingness to pay the fine believed that a higher percentage of others would pay the fine compared to those who chose to go to court, (M=73.1% vs. M=58.7%, t(1018)=18.0, p<0.001, d=1.27, 95% CI=[1.11, 1.42]. This pattern of data suggests that our results also replicated the original effect.

*STUDY 16*. In the study of Tversky & Kahneman (1981) on the effect of framing on decision-making, 181 individuals were surveyed about a situation where they were tasked with buying two items: a cheap item (priced at $15) and an expensive item (priced at $125) at a store. 93 of the individuals were assigned to the condition where the cheap item could be purchased for $5 less at the store’s other branch, a 20-minute drive away. In the sample, 88 of the individuals

**Figure S3**. Subjects’ answers when asked, for the supermarket scenario in the study of Ross et al. (1977), whether they would sign a release agreement for video footage on them to be used for a supermarket commercial. The “correct answer” of signing the release was robust to reversing the order of answer choices.


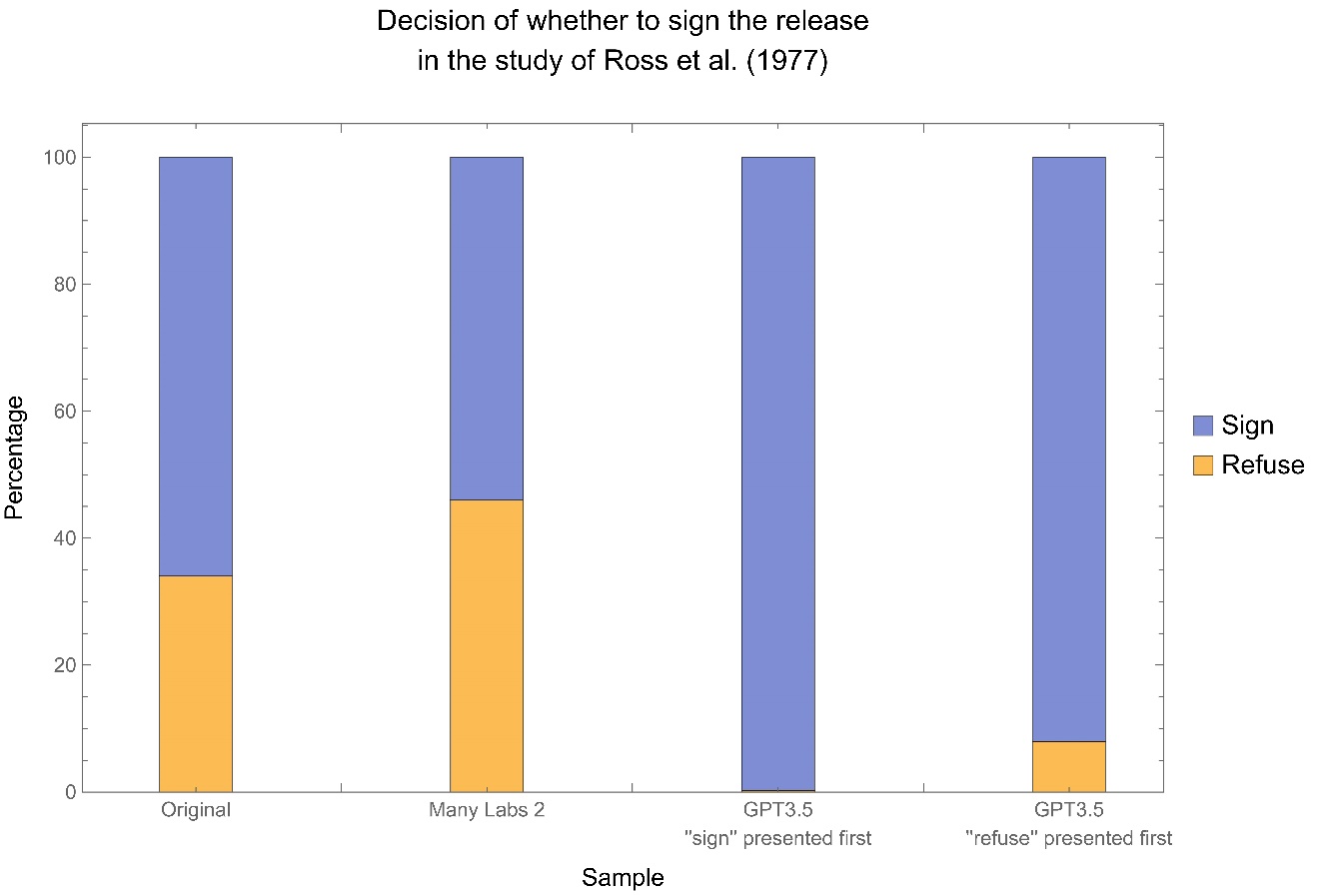


were assigned to the condition where the expensive item could be purchased for the same amount less at the other store. Consequently, the hypothetical cost saving was equal in both conditions. Individuals tended to decide to go to the other store more when the cost saving applied to the cheap item (68%) than when it applied to the expensive item (29%). This difference was statistically significant (z=5.14, p=7.4e^−7^, OR=4.96, 95% CI=[2.55, 9.90]), resulting in the finding that the decision of whether to go to the distant store was affected by the base cost of the discounted item rather than just the total discount. The Many Labs 2 sample (N=7,228) replicated this with inflation-adjusted prices and different item types. They successfully replicated Tversky and Kahneman’s finding, in that individuals tended to decide to go to the other store more when the cost saving applied to the cheap item (49%) than when it applied to the expensive item (32%). This difference was statistically significant (p=1.01e^−50^, d=0.40, 95% CI=[0.35, 0.45]; OR=2.06, 95% CI=[1.87, 2.27]), although the effect size is smaller than the original. However, in our GPT replication with Tversky and Kahneman’s original study (N=1,040, with 520 in each condition), all 100% of runs in both conditions answered that they would travel to the far-away store for the discount; see Figure S4. This unexpected uniformity of answers made the statistic we planned to analyse unconstructable, although we can say that our GPT sample’s answers did not reveal any evidence of the cognitive bias where decision-making is affected by the base cost of the discounted item rather than just the total discount.

We conducted an unplanned study with the reverse order of answer choices for the question pertaining to the aforementioned scenario (N=1,040, with 520 in each condition). However, the “correct answer” was robust to this order change. In the condition where the cheap item was discounted, all 100% runs still answered that they would travel to the distant store for the discount. In the condition where the expensive item was discounted, an overwhelming 99.8% of surveyed GPT runs (a total of 1,029) still responded that they would travel to the distant store, whereas only 0.2% of GPT runs (a total of just one) responded that they would remain at the current store and buy the items undiscounted. See Figure S4 for a visual summary.

*STUDY 18.* The paper by Risen & Gilovich (2008) surveyed 120 individuals to examine whether people thought that tempting fate increased the likelihood of bad outcomes, via a survey pertaining to a hypothetical classroom setting. In their original sample, the likelihood of being called upon was seen as higher when the student in question had tempted fate (M=3.43, SD=2.34) than if they had not and had prepared for the class (M=2.53, SD=2.24), t(116)=2.15, p=0.034, d=0.39, 95% CI=[0.03, 0.75]. The Many Labs 2 sample (N=8,000) replicated this effect. The likelihood of being called upon was seen as higher when the student in question had tempted fate (M=4.58, SD=2.44) than if they had prepared for the class (M=4.14, SD=2.45), t(7998)=8.08, p<0.001, d=0.18, 95% CI=[0.14, 0.22]. However, in the total sample (N=1,037) of our study, we find that the likelihood of being called on was judged as higher when the text mentioned having prepared for the class (M=4.75, SD=1.52) compared to when the text mentioned the subject having tempted fate (M=1.99, SD=0.42), t(1035)=40.0, p<0.001, d=2.49, 95% CI=[2.29, 2.68]. This is a very large effect in the opposite direction of both the original finding and the replication.

*STUDY 21.* Hsee (1998) surveyed 83 individuals to provide early evidence for the less-is-better effect. In their original sample, the less expensive scarf gift (M=5.63) was seen as more generous than the more expensive coat gift (M=5.00), t(82)=3.13, p=0.002, d=0.69, 95% CI=[0.24, 1.13], when the cheaper scarf gift was a higher-priced item in its respective category compared to a lower-priced item in the expensive category of coats. The Many Labs 2 sample (N=7,646) replicated this finding; participants in the scarf condition considered their gift more

**Figure S4**. Subjects’ answers to the survey of Tversky and Kahneman (1981) when asked whether—in a situation of needing to buy a cheap item and an expensive item—they would make a trip to a far-away store to buy one of the two at a fixed discount. The “correct answer” of making the trip was robust to reversing the order of answer choices.

**
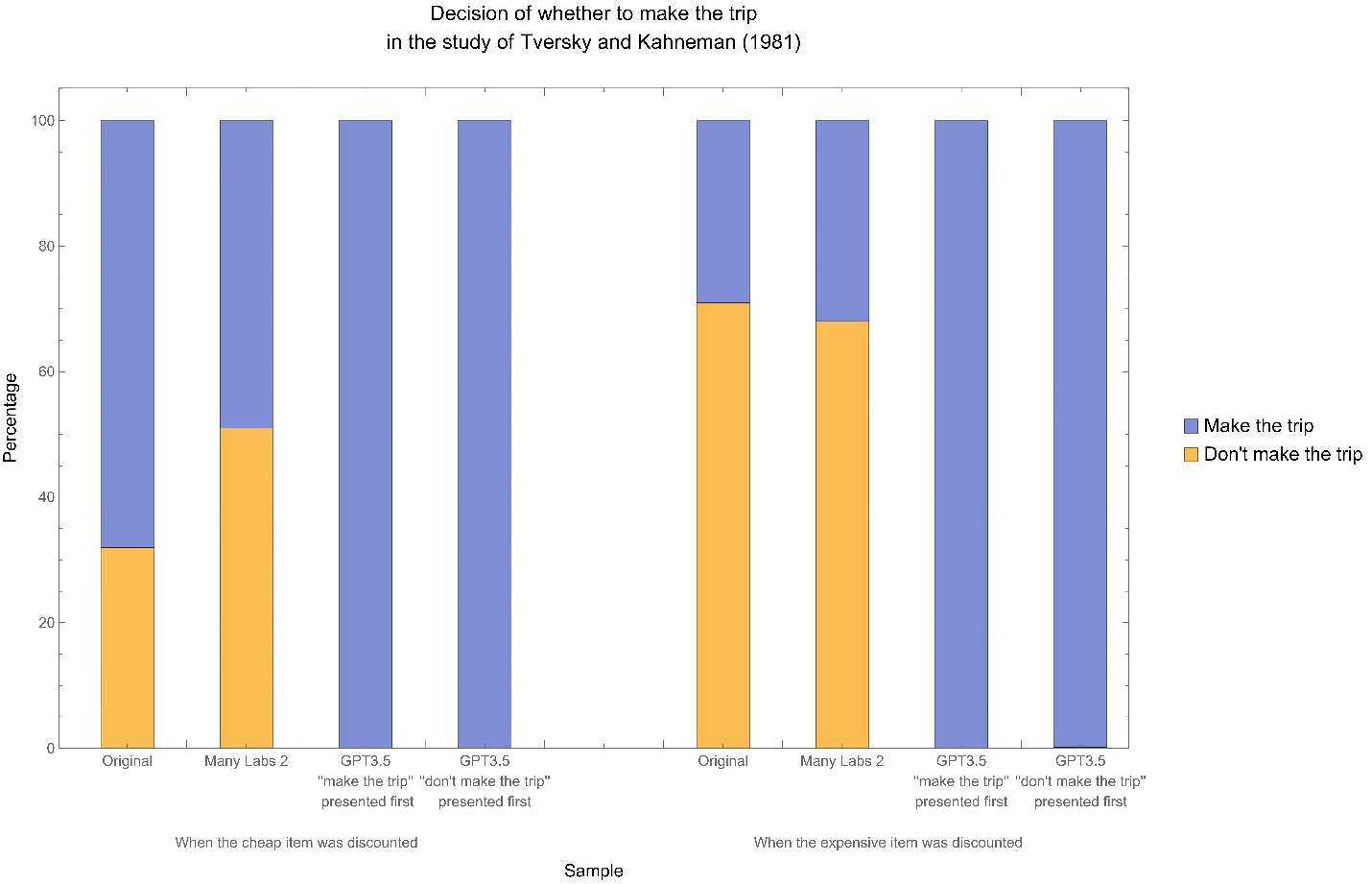
**

generous (M=5.50, SD=0.89) than did participants in the coat condition, (M=4.61, SD=1.34), t(6569.67)=34.20, p<0.001, d=0.78, 95% CI=[0.74, 0.83]. In our sample (N=1,040), we find that those in the scarf condition saw the gift giver as significantly more generous (M=5.97, SD=0.16) than those in the coat condition (M=3.99, SD=0.26), t(1038)=149, p<0.001, d=9.25, 95% CI=[8.67, 9.82]. This again successfully replicates the original finding, albeit with an extremely large effect size.

*STUDY 24*. Schwarz et al. (1991) surveyed 100 individuals and found that participants who were asked about their life satisfaction with respect to their relationship specifically before being asked about their life satisfaction in general exhibited a high correlation between their two responses (r=0.67, p<0.05), whereas this correlation was markedly weaker when the two questions were presented in reverse (r=0.32, p<0.05). The difference between the two correlations was statistically significant, z=2.32, p<0.01, q=0.48, 95% CI=[0.07, 0.88]. Many Labs 2 did not replicate this effect, and in fact found the opposite effect. Asking participants about their life satisfaction with respect to their relationship before being asked about their life satisfaction in general resulted in a lower correlation between the two responses (r=0.38) than the other way around (r=0.44). The opposite-direction difference between the correlations was significant, z=-3.03, p=0.002, q=-0.07, 95% CI=[-0.12, -0.02]. In our total sample (N=1,040), we find that the correlation between the two questions was r=-0.446, p=0.001 for when the general question was asked first, and r=-0.490, p<0.001 for when the specific question was asked first. Using the Fisher’s r-to-z transformation (Weiss, 2011), we find that this difference was not significant, z=0.906, p=0.365, q=0.056, 95% CI=[-0.066, 0.178]. This is a wholly different pattern of results than both the original study and the Many Labs 2 replication, meaning that we did not replicate the effect in our GPT sample.

*STUDY 25*. Shafir (1994) had 170 participants make decisions between awarding or denying custody to either an average or an extreme parent. In the original sample, participants were more likely to both award (64%) and deny (55%) custody to the parent described with extreme characteristics than they were to the parent described with average characteristics. The sum of these probabilities (119%) was significantly higher than the expected quantity of 100% that one would expect if awarding and denying were complementary, z=2.48, p=0.013, d=0.35, 95% CI=[-0.04, 0.68]. This result indicated that negative features were weighted more heavily than positive ones in the case of rejections and vice versa. The Many Labs 2 sample (N=7,901) did not replicate this effect and in fact provided evidence in favour of an effect in the opposite direction. Participants were less likely to both award (45.5%) and deny (47.6%) custody to the parent described with extreme characteristics, and the sum of these probabilities (93%) was significantly lower than the expected quantity 100%, z=-6.10, p<0.001, d=-0.13, 95% CI=[-0.18, -0.09]. In our sample (N=857), we find that GPT3.5 runs were both more likely to award (95.8%) and deny (100%) custody to the extreme parent than the average parent. Via a logit calculation (Wilson, n.d.), we compute that the sum of these probabilities (195.8%) was significantly higher than the expected quantity of 100%, z=5.11, p<0.001, d=2.11, 95% CI=[1.56, 2.67]. This replicates the original finding that the Many Labs 2 data did not replicate, albeit with a very high effect size.

*STUDY 27.* The original finding by Knobe (2003), which surveyed 78 individuals, was that harmful side effects were judged as more intentional than helpful ones. In the original sample, 82% of participants who were told about an agent (a company’s board chairman) whose decision brought about a harmful side effect said the agent did so intentionally, while 23% of participants in the analogous helpful-side-effect condition said that the agent brought about the side effect intentionally, χ^2^(1, N=78)=27.2, p<0.001, d=1.45, 95% CI=[0.79, 2.77]. This was replicated by Many Labs 2, which used a seven-point scale of intentionality rather than a yes-no scale. Also, in the Many Labs 2 sample, those in the harmful-side-effect condition (M = 6.03, SD = 1.26) placed a higher degree of blame than the degree of praise (M = 2.54, SD = 1.60) in the helpful-side-effect condition, t(7553.82) = 108.15, p < 1.68e^−305^, d = 2.42, 95% CI = [2.36, 2.48]. Our sample (N=1,040) consisted of 520 surveyed runs in each condition, using the original version of the survey that used a yes-no scale as well as the seven-point scale for blame/praise. However, the question on rating blame/praise on the seven-point scale saw “correct answers” for both conditions. In the positive-side-effect condition, 99.2% of surveyed GPT runs (a total of 516) described the positive side effect as deserving of a degree seven of praise, or “a lot of praise”; 0.2% of them (a total of just one) described it as deserving of a degree six of praise; and 0.6% of them (a total of 3) described it as deserving of a degree one of praise, or “no praise.” In the negative-side-effect condition, 100% of surveyed GPT runs described the negative side effect as deserving of a degree seven of praise, or “a lot of praise.” Our pre-registered analysis plan was made unsuitable by the unexpected uniformity of GPT3.5’s answers.

We conducted an unplanned and exploratory follow-up study with the reverse order of answer choices for the question pertaining to how much blame/praise is deserved (N=1,040, with 520 in each condition). Specifically, we presented the answer choices in reverse order, from ‘A Lot of Praise’ to ‘No Praise.’ In the positive-side-effect condition, the reversal of order resulted in only 3.5% of surveyed GPT runs giving the original “correct answer” of ‘A Lot of Praise’ (a total of just 18 runs), 0.2% of GPT runs answering with the second-highest level of praise (a total of just one GPT run), and the remaining 96.3% of GPT runs answering with ‘No Praise’ (a total of 501 runs). The original “correct answer” of ‘A Lot of Praise’ did not replicate. However, in the negative-side-effect condition, the reversal of order resulted in 93.7% of surveyed GPT runs giving the original “correct answer” of ‘A Lot of Praise’ (a total of 487 runs), 0.2% of runs responding with the second-highest degree of praise (a total of just one run), and 6.2% of runs responding “No Praise (a total of just 32 runs). The original “correct answer” of ‘A Lot of Blame’ successfully replicated after reversing the order of answer choices.

For a visual plot of the Cohen’s d effect sizes and the corresponding 95% confidence intervals corresponding to the relevant subset of our studies, see Figure S5.

We conducted another exploratory study as a follow-up. The purpose of this second follow-up was to test one of our design trade-offs, which is that we used exclusively temperature as a source of variation in output. As helpfully pointed out by two anonymous reviewers, one reason for the “correct answer” effect may be due to the prompt not including any demographic information, thus artificially restricting variation of the LLM responses compared with a diverses et of human participants. To investigate this concern, we replicated one of the studies that showed a “correct answer” effect to address this concern. Specifically, we re-ran Study 11’s (Hauser et al. 2007) condition, where participants are asked to evaluate the moral permissibility of pushing a large man in front of a trolley to save the lives of five others. In this follow-up, we added a randomly selected combination of demographic characteristics to the prompt, instructing the LLM to “respond as a…” The categories that we randomly selected this prompt addition from were gender (male, female), age (20, 30, …, 70), religion (Christianity, Islam, Judaism), ethnicity (Black, White, Asian, Hispanic), and education (High School, College, Advanced Degree). The rest of the prompt remained the same for the condition that we were testing. We collected a total of 982 responses. There were 384 unique combinations of demographic variables (out of a maximum of 432 combinations). Across all combinations, we found strong evidence for the correct answer effect, as 100% of responses indicated that shoving the large man on the tracks to save five

**Figure S5**. Cohen’s d effect sizes (with 95% confidence intervals) for the original study, the Many Labs 2 replication, and our GPT3.5 re-replication. The figure excludes the study of Inbar et al. (2009) on whether disgust sensitivity predicts homophobia and the study of Schwarz et al. (1991) on the effects of assimilation and contrast in sequences of questions, because the original studies used Cohen’s q instead of Cohen’s d. The figure also excludes studies for which the effect size is too large to plot with visual convenience (greater than 3.0). This excluded us from plotting the unprecedentedly high effect size of d=9.25 for our replication of the study of Hsee (1998) on the less-is-better effect, due to the “correct answer” effect of GPT3.5 responding to the inputs of different conditions with much more predeterminedly different answers than did human subjects.


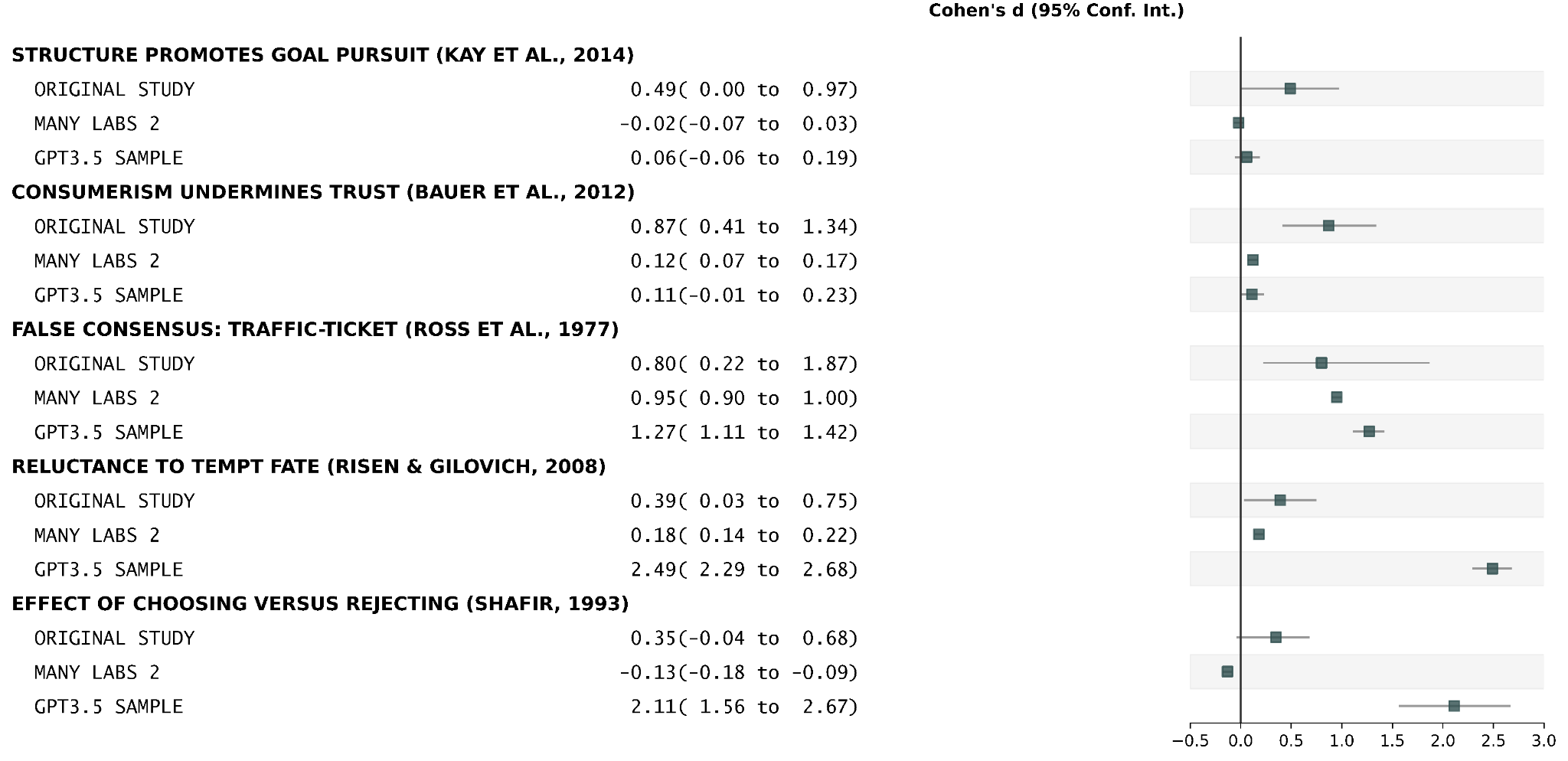


others was morally impermissible. This provides evidence that our initial result is unlikely to be primarily explained by the source of variation coming from the temperature setting.

**References**

Bauer, M. A., Wilkie, J. E., Kim, J. K., & Bodenhausen, G. V. (2012). Cuing consumerism: Situational materialism undermines personal and social well-being. *Psychological Science*, 23(5), 517-523. <https://doi.org/10.1177/0956797611429579>

Graham, J., Haidt, J., & Nosek, B. A. (2009). Liberals and conservatives rely on different sets of moral foundations. *Journal of Personality and Social Psychology*, 96(5), 1029-1046. https://doi.org/10.1037/a0015141

Hauser, M., Cushman, F., Young, L., Kang‐Xing Jin, R., & Mikhail, J. (2007). A dissociation between moral judgments and justifications. *Mind & Language*, 22(1), 1-21. <https://psycnet.apa.org/doi/10.1111/j.1468-0017.2006.00297.x>

Hsee, C. K. (1998). Less is better: When low‐value options are valued more highly than high‐value options. *Journal of Behavioral Decision Making*, 11(2), 107-121. <https://doi.org/10.1002/(SICI)1099-0771(199806)11:2%3C107::AID-BDM292%3E3.0.CO;2-Y>

Inbar, Y., Pizarro, D. A., Knobe, J., & Bloom, P. (2009). Disgust sensitivity predicts intuitive disapproval of gays. *Emotion*, 9(3), 435-439. https://doi.org/10.1037/a0015960

Kay, A. C., Laurin, K., Fitzsimons, G. M., & Landau, M. J. (2014). A functional basis for structure-seeking: Exposure to structure promotes willingness to engage in motivated action. *Journal of Experimental Psychology: General*, 143(2), 486-491. https://doi.org/10.1037/a0034462

Klein, R. A., Vianello, M., Hasselman, F., Adams, B. G., Adams Jr., R. B., Alper, S., Aveyard, M., Axt, J. R., Babalola, M. T., Bahník, Š., Batra, R., Berkics, M., Bernstein, M. J., Berry D. R., Bialobrzeska, O., Binan, E. D., Bocian, K., Brandt, M. J., Busching, R., ... & Sowden, W. (2018). Many Labs 2: Investigating variation in replicability across samples and settings. *Advances in Methods and Practices in Psychological Science*, 1(4), 443-490. <https://doi.org/10.1177/2515245918810225>

Knobe, J. (2003). Intentional action and side effects in ordinary language. *Analysis*, 63, 190–193. <https://doi.org/10.1111/1467-8284.00419>

OpenAI (2023a). *Completions*. OpenAI. Retrieved February 8, 2023, from <https://platform.openai.com/docs/api-reference/completions>

OpenAI (2023b). *Pricing*. OpenAI. Retrieved March 2, 2023, from <https://openai.com/api/pricing/>

Park, P. S., Schoenegger, P., Zhu, C, & Maier, M.. (2023). AI psychology [Pre-registration, source code, and data]. Open Science Framework. Retrieved February 13, 2023 from <https://osf.io/dzp8t/?view_only=45fff3953884443d81b628cdd5d50f7a>

R Core Team (2021). *R: A Language and environment for statistical computing*. (Version 4.1) [Computer software]. Retrieved from<https://cran.r-project.org>. (R packages retrieved from MRAN snapshot 2022-01-01).

Risen, J. L., & Gilovich, T. (2008). Why people are reluctant to tempt fate. *Journal of Personality and Social Psychology*, 95(2), 293-307. <https://doi.org/10.1037/0022-3514.95.2.293>

Ross, L., Greene, D., & House, P. (1977). The “false consensus effect”: An egocentric bias in social perception and attribution processes. *Journal of Experimental Social Psychology*, 13(3), 279-301. <https://doi.org/10.1016/0022-1031(77)90049-X>

Rottenstreich, Y., & Hsee, C. K. (2001). Money, kisses, and electric shocks: On the affective psychology of risk. *Psychological Science*, 12(3), 185-190. <https://doi.org/10.1111/1467-9280.00334>

Schwartz, S. H. (2003). A proposal for measuring value orientations across nations. *Questionnaire Package of the European Social Survey*, 259(290), 259-319.

Schwarz, N., Strack, F., & Mai, H. P. (1991). Assimilation and contrast effects in part-whole question sequences: A conversational logic analysis. *Public Opinion Quarterly*, 55(1), 3-23. <https://doi.org/10.1086/269239>

Shafir, E. (1993). Choosing versus rejecting: Why some options are both better and worse than others. *Memory & Cognition*, 21(4), 546-556. <https://doi.org/10.3758/bf03197186>

The jamovi project (2022). *jamovi*. (Version 2.3) [Computer software]. Retrieved from<https://www.jamovi.org>.

Tversky, A., & Kahneman, D. (1981). The framing of decisions and the psychology of choice. *Science*, 211, 453–458. <https://doi.org/10.1126/science.7455683>

Weiss, B. A. (2011). Fisher’s r-to-Z transformation calculator to compare two independent samples [Computer software]. Available from <https://blogs.gwu.edu/weissba/teaching/calculators/fishers-z-transformation/>.

Wilson, D. B. (n.d.). Outcome frequency [Computer software]. Practical Meta-Analysis Effect Size Calculator. Available from <https://www.campbellcollaboration.org/escalc/html/EffectSizeCalculator-SMD9.php>.
